# Supplementary material for: Objectively measured physical activity levels and adherence to physical activity guidelines in people with multimorbidity—A systematic review and meta-analysis
Source: PLoS One. 2022 Oct 12;17(10):e0274846. doi: 10.1371/journal.pone.0274846 (PMC9555650; doi:10.1371/journal.pone.0274846)
Supplement: S2 File — (PDF) [file pone.0274846.s002.pdf]

S2 Search strategy for Medline via OVID

1 accelerometers*.mp.

2 pedomet*.mp.

3 motion sensor*.mp.

4 multisensor*.mp.

5 multi-sensor*.mp.

6 direct observation*.mp.

7 direct measurement*.mp.

8 objective measurement*.mp.

9 ((arm band or wrist band or heart rate or heart-rate or heart rhythm or heart-rhythm or step or measuring or electronic* or mechanic* or portable or wearable or motion or physical activity or fitness or activity) adj3 (track* or device* or sensor* or detector* or monitor* or count*)).mp.

10 ((cell* or smart* or mobile or android or internet or web) adj3 (comput* or device or app* or phone)).mp.

11 1 or 2 or 3 or 4 or 5 or 6 or 7 or 8 or 9 or 10

12 Physical activity.mp.

13 exp Sports/

14 Physical Fitness/

15 fitness.mp.

16 aerobics.mp.

17 Exercise/

18 Exercis*.mp.

19 exp Exercise Therapy/

20 Exercise Therapy.mp.

21 Locomotion/

22 exp Physical Therapy Modalities/

23 physiotherapy.mp.

24 physical therapy.mp.

25 Motor Activity/

26 exp Walking/

27 walking.mp.

28 running.mp.

29 exp Running/

30 jogging.mp.

31 exp Bicycling/

32 cycling.mp.

33 exp Swimming/

34 swimming.mp.

35 exp Gymnastics/

36 gymnastic*.mp.

37 exp Dancing/

38 12 or 13 or 14 or 17 or 18 or 19 or 20 or 21 or 22 or 25 or 26 or 27 or 28 or 29 or 31 or 33 or 34 or 35 or 36 or 37

39 exp multimorbidity/

40 multimorbid*.mp.

41 multi-morbid*.mp.

42 exp multiple chronic conditions/

43 exp comorbidity/

44 comorbid*.mp.

45 co-morbid*.mp.

46 exp noncommunicable diseases/

47 ((concurrent or simultaneous or dual or multi or multiple or pluri or poly or chronic* or coexist* or co-exist* or co-occur* or cooccur*) adj3 (condition* or disease* or illness* or disorder* or morbidit* or patholog* or diagnos* or syndrome* or health problem*)).mp.

48 39 or 40 or 41 or 42 or 43 or 44 or 45 or 46 or 47

49 exp myocardial ischemia/

50 myocardial ischemia.mp.

51 exp coronary artery disease/

52 coronary artery disease.mp.

53 exp coronary disease/

54 coronary disease.mp.

55 exp myocardial infarction/

56 myocardial infarction.mp.

57 exp angina pectoris/

58 angina pectoris.mp.

59 exp heart failure/

60 heart failure.mp.

61 HFNEF.mp.

62 HFPEF.mp.

63 HFREF.mp.

64 "HF NEF".mp.

65 "HF PEF".mp.

66 "HF REF".mp.

67 exp heart diseases/

68 heart diseases.mp.

69 coronary artery bypass.mp.

70 49 or 50 or 51 or 52 or 53 or 54 or 55 or 56 or 57 or 58 or 59 or 60 or 61 or 62 or 63 or 64 or 65 or 66 or 67 or 68 or 69

71 exp pulmonary disease, chronic obstructive/

72 exp COPD/

73 COPD.mp.

74 exp pulmonary emphysema/

75 pulmonary emphysema.mp.

76 exp COAD/

77 COAD.mp.

78 exp Bronchitis, Chronic/

79 chronic bronchitis.mp.

80 chronic obstructive lung disease.mp.

81 71 or 72 or 73 or 74 or 75 or 76 or 77 or 78 or 79 or 80

82 exp hypertension/

83 hypertens*.mp.

84 high blood pressure.mp.

85 exp blood pressure/

86 82 or 83 or 84 or 85

87 exp depression/

88 depression.mp.

89 exp dysthymic disorder/

90 dysthymi*.mp.

91 ((dysthymic or affect*) adj2 (disorder* or symptom*)).mp.

92 87 or 88 or 89 or 90 or 91

93 exp anxiety/

94 anxiety.mp.

95 exp anxiety disorders/

96 93 or 94 or 95

97 exp diabetes mellitus/

98 diabetes mellitus.mp.

99 exp diabetes mellitus, type 2/

100 Type 2 diab*.mp.

101 Type II diab*.mp.

102 Non-Insulin-Dependent Diabetes Mellitus.mp.

103 NIDDM.mp.

104 impaired glucose toleranc*.mp.

105 exp glucose intolerance/

106 exp blood glucose/

107 97 or 98 or 99 or 100 or 101 or 102 or 103 or 104 or 105 or 106

108 exp osteoarthritis/

109 osteoarthritis.mp.

110 osteoarthrit*.mp.

111 osteoarthros*.mp.

112 108 or 109 or 110 or 111

113 exp Spinal Stenosis/

114 (spin* adj5 stenos*).mp.

115 (lumbar adj5 stenos*).mp.

116 (neuro* adj2 claud*).mp.

117 lumbar radicular pain.mp.

118 exp Cauda Equina/

119 cauda equina.mp.

120 exp Spinal Osteophytosis/

121 spinal osteophytosis.mp.

122 exp Spondylosis/

123 spondylos*.mp.

124 exp Spondylolisthesis/

125 spondylolisthesis.mp.

126 exp Low Back Pain/

127 (low* adj5 back adj5 pain).mp.

128 113 or 114 or 115 or 116 or 117 or 118 or 119 or 120 or 121 or 122 or 123 or 124 or 125 or 126 or 127

129 70 and (81 or 86 or 92 or 96 or 107 or 112 or 128)

130 81 and (86 or 92 or 96 or 107 or 112 or 128)

131 86 and (92 or 96 or 107 or 112 or 128)

132 92 and (96 or 107 or 112 or 128)

133 96 and (107 or 112 or 128)

134 107 and (112 or 128)

135 112 and 128

136 11 and 38 and (48 or 129 or 130 or 131 or 132 or 133 or 134 or 135)

137 (Animals/ or Models, Animal/ or Disease Models, Animal/) not Humans/

138 ((animal or animals or canine* or dog or dogs or feline or hamster* or lamb or lambs or mice or monkey or monkeys or mouse or murine or pig or pigs or piglet* or porcine or primate* or rabbit* or rats or rat or rodent* or sheep* or veterinar*) not (human* or patient*)).ti,kf,jw.

139 137 or 138

140 136 not 139
