# Supplementary material for: Objectively measured physical activity levels and adherence to physical activity guidelines in people with multimorbidity—A systematic review and meta-analysis
Source: PLoS One. 2022 Oct 12;17(10):e0274846. doi: 10.1371/journal.pone.0274846 (PMC9555650; doi:10.1371/journal.pone.0274846)
Supplement: S3 File — (PDF) [file pone.0274846.s003.pdf]

S3 Search strategy for EMBASE via OVID

1 accelerometer*.mp.

2 pedomet*.mp.

3 motion sensor*.mp.

4 multisensor*.mp.

5 multi-sensor*.mp.

6 direct observation*.mp.

7 direct measurement*.mp.

8 objective measurement*.mp.

9 ((arm band or wrist band or heart rate or heart-rate or heart rhythm or heart-rhythm or step or measuring or electronic* or mechanic* or portable or wearable or motion or physical activity or fitness or activity) adj3 (track* or device* or sensor* or detector* or monitor* or count*)).mp.

10 ((cell* or smart* or mobile or android or internet or web) adj3 (comput* or device or app* or phone)).mp.

11 1 or 2 or 3 or 4 or 5 or 6 or 7 or 8 or 9 or 10

12 Physical activity.mp.

13 exp Sports/

14 Physical Fitness/

15 fitness.mp.

16 aerobics.mp.

17 Exercise/

18 Exercis*.mp.

19 exp Exercise Therapy/

20 Exercise Therapy.mp.

21 Locomotion/

22 exp Physical Therapy Modalities/

23 physiotherapy.mp.

24 physical therapy.mp.

25 Motor Activity/

26 exp Walking/

27 walking.mp.

28 running.mp.

29 exp Running/

30 jogging.mp.

31 exp Bicycling/

32 cycling.mp.

33 exp Swimming/

34 swimming.mp.

35 exp Gymnastics/

36 gymnastic*.mp.

37 exp Dancing/

38 12 or 13 or 14 or 17 or 18 or 19 or 20 or 21 or 22 or 25 or 26 or 27 or 28 or 29 or 31 or 33 or 34 or 35 or 36 or 37

39 exp multimorbidity/

40 multimorbid*.mp.

41 multi-morbid*.mp.

42 exp multiple chronic conditions/

43 exp comorbidity/

44 comorbid*.mp.

45 co-morbid*.mp.

46 exp noncommunicable diseases/

47 ((concurrent or simultaneous or dual or multi or multiple or pluri or poly or chronic* or coexist* or co-exist* or co-occur* or cooccur*) adj3 (condition* or disease* or illness* or disorder* or morbidit* or patholog* or diagnos* or syndrome* or health problem*)).mp.

48 39 or 40 or 41 or 42 or 43 or 44 or 45 or 46 or 47

49 exp myocardial ischemia/

50 myocardial ischemia.mp.

51 exp coronary artery disease/

52 coronary artery disease.mp.

53 exp coronary disease/

54 coronary disease.mp.

55 exp myocardial infarction/

56 myocardial infarction.mp.

57 exp angina pectoris/

58 angina pectoris.mp.

59 exp heart failure/

60 heart failure.mp.

61 HFNEF.mp.

62 HFPEF.mp.

63 HFREF.mp.

64 "HF NEF".mp.

65 "HF PEF".mp.

66 "HF REF".mp.

67 exp heart diseases/

68 heart diseases.mp.

69 coronary artery bypass.mp.

70 49 or 50 or 51 or 52 or 53 or 54 or 55 or 56 or 57 or 58 or 59 or 60 or 61 or 62 or 63 or 64 or 65 or 66 or 67 or 68 or 69

71 exp pulmonary disease, chronic obstructive/

72 exp COPD/

73 COPD.mp.

74 exp pulmonary emphysema/

75 pulmonary emphysema.mp.

76 COAD.mp.

77 exp Bronchitis, Chronic/

78 chronic bronchitis.mp.

79 chronic obstructive lung disease.mp.

80 71 or 72 or 73 or 74 or 75 or 76 or 77 or 78 or 79

81 exp hypertension/

82 hypertens*.mp.

83 high blood pressure.mp.

84 exp blood pressure/

85 81 or 82 or 83 or 84

86 exp depression/

87 depression.mp.

88 exp dysthymic disorder/

89 dysthymi*.mp.

90 ((dysthymic or affect*) adj2 (disorder* or symptom*)).mp.

91 86 or 87 or 88 or 89 or 90

92 exp anxiety/

93 anxiety.mp.

94 exp anxiety disorders/

95 92 or 93 or 94

96 exp diabetes mellitus/

97 diabetes mellitus.mp.

98 exp diabetes mellitus, type 2/

99 Type 2 diab*.mp.

100 Type II diab*.mp.

101 Non-Insulin-Dependent Diabetes Mellitus.mp.

102 NIDDM.mp.

103 impaired glucose toleranc*.mp.

104 exp glucose intolerance/

105 exp blood glucose/

106 96 or 97 or 98 or 99 or 100 or 101 or 102 or 103 or 104 or 105

107 exp osteoarthritis/

108 osteoarthritis.mp.

109 osteoarthrit*.mp.

110 osteoarthros*.mp.

111 107 or 108 or 109 or 110

112 exp Spinal Stenosis/

113 (spin* adj5 stenos*).mp.

114 (lumbar adj5 stenos*).mp.

115 (neuro* adj2 claud*).mp.

116 lumbar radicular pain.mp.

117 exp Cauda Equina/

118 cauda equina.mp.

119 exp Spinal Osteophytosis/

120 spinal osteophytosis.mp.

121 exp Spondylosis/

122 spondylos*.mp.

123 exp Spondylolisthesis/

124 spondylolisthesis.mp.

125 exp Low Back Pain/

126 (low* adj5 back adj5 pain).mp.

127 112 or 113 or 114 or 115 or 116 or 117 or 118 or 119 or 120 or 121 or 122 or 123 or 124 or 125 or 126 (102663)

128 70 and (80 or 85 or 91 or 95 or 106 or 111 or 127)

129 80 and (85 or 91 or 95 or 106 or 111 or 127)

130 85 and (91 or 95 or 106 or 111 or 127)

131 91 and (95 or 106 or 111 or 127)

132 95 and (106 or 111 or 127)

133 106 and (111 or 127)

134 111 and 127

135 11 and 38 and (48 or 128 or 129 or 130 or 131 or 132 or 133 or 134)

136 (animal or animals or canine* or dog or dogs or feline or hamster* or lamb or lambs or mice or monkey or monkeys or mouse or murine or pig or pigs or piglet* or porcine or primate* or rabbit* or rats or rat or rodent* or sheep* or veterinar*).ti,kw,dq,jx. not (human* or patient*).mp.

137 (exp animal/ or exp juvenile animal/ or adult animal/ or animal cell/ or animal tissue/ or nonhuman/ or animal experiment/ or animal model/) not human/

138 136 or 137

139 135 not 138
