# Supplementary material for: Objectively measured physical activity levels and adherence to physical activity guidelines in people with multimorbidity—A systematic review and meta-analysis
Source: PLoS One. 2022 Oct 12;17(10):e0274846. doi: 10.1371/journal.pone.0274846 (PMC9555650; doi:10.1371/journal.pone.0274846)
Supplement: S4 File — (PDF) [file pone.0274846.s004.pdf]

S4 Search strategy CIHNAL via EBSCOhost

S132	S130 NOT S131
S131	TI ((animal or animals or canine* or dog or dogs or feline or hamster* or lamb or lambs or mice or monkey or monkeys or mouse or murine or pig or pigs or piglet* or porcine or primate* or rabbit* or rats or rat or rodent* or sheep*) NOT (human* or patient*)
S130	S11 AND S37 AND (S42 OR S123 OR S124 OR S125 OR S126 OR S127 OR S128 OR S129)
S129	S104 AND S122
S128	S100 AND (S104 OR S122)
S127	S87 AND (S100 OR S104 OR S122)
S126	S83 AND (S87 OR S100 OR S104 OR S122)
S125	S77 AND (S83 OR S87 OR S100 OR S104 OR S122)
S124	S73 AND (S77 OR S83 OR S87 OR S100 OR S104 OR S122)
S123	S65 AND (S73 OR S77 OR S83 OR S87 OR S100 OR S104 OR S122)
S122	S105 OR S106 OR S107 OR S108 OR S109 OR S110 OR S111 OR S112 OR S113 OR S114 OR S115 OR S116 OR S117 OR S118 OR S119 OR S120 OR S121
S121	(TI low* OR AB low* OR TW low*) n5 (TI back OR AB back OR TW back) n5 (TI pain OR AB pain OR TW pain)
S120	(MH "Low Back Pain")
S119	TI Spondylolisthesis OR AB Spondylolisthesis OR TW Spondylolisthesis
S118	(MH "Spondylolisthesis")
S117	TI Spondylos* OR AB Spondylos* OR TW Spondylos*
S116	(MH "Spondylosis+")
S115	TI Spinal Osteophytosis OR AB Spinal Osteophytosis OR TW Spinal Osteophytosis
S114	(MH "Spinal Osteophytosis+")
S113	TI cauda equina OR AB cauda equina OR TW cauda equina
S112	(MH "Cauda Equina Syndrome")
S111	(MH "Cauda Equina")
S110	TI lumbar radicular pain OR AB lumbar radicular pain OR TW lumbar radicular pain
S109	(TI neuro* OR AB neuro* OR TW neuro*) n2 (TI claud* OR AB claud* OR TW claud*)
S108	(TI lumbar OR AB lumbar OR TW lumbar) n5 (TI stenos* OR AB stenos* OR TW stenos*)
S107	(TI spin* OR AB spin* OR TW spin*) n5 (TI stenos* OR AB stenos* OR TW stenos*)
S106	TI Spinal Stenosis OR AB Spinal Stenosis OR TW Spinal Stenosis
S105	(MH "Spinal Stenosis")
S104	S101 OR S102 OR S103

S103	TI osteoarthros* OR AB osteoarthros* OR TW osteoarthros*
S102	TI osteoarthrit* OR AB osteoarthrit* OR TW osteoarthrit*
S101	(MH "Osteoarthritis+")
S100	S88 OR S89 OR S90 OR S91 OR S92 OR S93 OR S94 OR S95 OR S96 OR S97 OR S98 OR S99
S99	(MH "Blood Glucose")
S98	TI glucose intolerance OR AB glucose intolerance OR TW glucose intolerance
S97	(MH "Glucose Intolerance")
S96	TI impaired glucose toleranc* OR AB impaired glucose toleranc* OR TW impaired glucose toleranc*
S95	TI NIDDM OR AB NIDDM OR TW NIDDM
S94	TI Non-Insulin-Dependent Diabetes Mellitus OR AB Non-Insulin-Dependent Diabetes Mellitus OR TW Non-Insulin-Dependent Diabetes Mellitus
S93	TI Type II diab* OR AB Type II diab* OR TW Type II diab*
S92	TI Type 2 diab* OR AB Type 2 diab* OR TW Type 2 diab*
S91	TI Diabetes Mellitus, Type 2 OR AB Diabetes Mellitus, Type 2 OR TW Diabetes Mellitus, Type 2
S90	(MH "Diabetes Mellitus, Type 2")
S89	TI diabetes mellitus OR AB diabetes mellitus OR TW diabetes mellitus
S88	(MH "Diabetes Mellitus+")
S87	S84 OR S85 OR S86
S86	TI anxiety disorder* OR AB anxiety disorder* OR TW anxiety disorder*
S85	TI anxiety OR AB anxiety OR TW anxiety
S84	(MH "Anxiety+")
S83	S78 OR S79 OR S80 OR S81 OR S82
S82	(TI dysthymic OR AB dysthymic OR TW dysthymic OR TI affect* OR AB affect* OR TW affect*) n2 (TI disorder* OR AB disorder* OR TW disorder* OR TI symptom* OR AB symptom* OR TW symptom*)
S81	TI dysthymi* OR AB dysthymi* OR TW dysthymi*
S80	(MH "Dysthymic Disorder")
S79	TI depression OR AB depression OR TW depression
S78	(MH "Depression+")
S77	S74 OR S75 OR S76
S76	TI high blood pressure OR AB high blood pressure OR TW
S75	TI hypertens* OR AB hypertens* OR TW hypertens*
S74	(MH "Hypertension+")

S73	S66 OR S67 OR S68 OR S69 OR S70 OR S71 OR S72
S72	(MH "Lung Diseases, Obstructive+")
S71	TI chronic bronchitis OR AB chronic bronchitis OR TW chronic bronchitis
S70	(MH "Bronchitis, Chronic")
S69	TI pulmonary emphysema OR AB pulmonary emphysema OR TW pulmonary emphysema
S68	(MH "Emphysema")
S67	TI chronic obstructive pulmonary disease OR AB chronic obstructive pulmonary disease OR TW chronic obstructive pulmonary disease
S66	(MH "Pulmonary Disease, Chronic Obstructive+")
S65	S43 OR S44 OR S45 OR S46 OR S47 OR S48 OR S49 OR S50 OR S51 OR S52 OR S53 OR S54 OR S55 OR S56 OR S57 OR S58 OR S59 OR S60 OR S61 OR S62 OR S63 OR S64
S64	TI coronary artery bypass OR AB coronary artery bypass OR TW coronary artery bypass
S63	(MH "Coronary Artery Bypass+")
S62	TI heart diseases OR AB heart diseases OR TW heart diseases
S61	(MH "Heart Diseases+")
S60	TI "HF REF" OR AB "HF REF" OR TW "HF REF"
S59	TI "HF PEF" OR AB "HF PEF" OR TW "HF PEF"
S58	TI "HF NEF" OR AB "HF NEF" OR TW "HF NEF"
S57	TI HFREF OR AB HFREF OR TW HFREF
S56	TI HFPEF OR AB HFPEF OR TW HFPEF
S55	TI HFNEF OR AB HFNEF OR TW HFNEF
S54	TI heart failure OR AB heart failure OR TW heart failure
S53	(MH "Heart Failure+")
S52	TI angina pectoris OR AB angina pectoris OR TW angina pectoris
S51	(MH "Angina Pectoris+")
S50	TI myocardial infarction OR AB myocardial infarction OR TW myocardial infarction
S49	(MH "Myocardial Infarction+")
S48	TI coronary disease OR AB coronary disease OR TW coronary disease
S47	(MH "Coronary Disease+")
S46	TI coronary artery disease OR AB coronary artery disease OR TW coronary artery disease
S45	(MH "Coronary Arteriosclerosis")
S44	TI myocardial ischemia OR AB myocardial ischemia OR TW myocardial ischemia
S43	(MH "Myocardial Ischemia+")

S42	S38 OR S39 OR S40 OR S41
S41	(TI concurrent OR AB concurrent OR TW concurrent OR TI simultaneous OR AB simultaneous OR TW simultaneous OR TI dual OR AB dual OR TW dual OR TI multi OR AB multi OR TW multi OR TI multiple OR AB multiple OR TW multiple OR TI pluri OR AB pluri OR TW pluri OR TI poly OR AB poly OR TW poly OR TI chronic* OR AB chronic* OR TW chronic* OR TI coexist* OR AB coexist* OR TW coexist* OR TI co-exist* OR AB co-exist* OR TW co-exist* OR TI co-occur* OR AB co-occur* OR TW co-occur* OR TI cooccur* OR AB cooccur* OR TW cooccur*) n3 (TI condition* OR AB condition* OR TW condition* OR TI disease* OR AB disease* OR TW disease* OR TI illness* OR AB illness* OR TW illness* OR TI disorder* OR AB disorder* OR TW disorder* OR TI morbidit* OR AB morbidit* OR TW morbidit* OR TI patholog* OR AB patholog* OR TW patholog* OR TI diagnos* OR AB diagnos* OR TW diagnos* OR TI syndrome* OR AB syndrome* OR TW syndrome* OR TI health problem*) OR AB health problem* OR TW health problem*)
S40	TI multimorbid* OR AB multimorbid* OR TW multimorbid* OR TI multi-morbid* OR AB multi-morbid* OR TW multi-morbid* OR TI comorbid* OR AB comorbid* OR TW comorbid* OR TI co-morbid* OR AB co-morbid* OR TW co-morbid*
S39	(MH "Comorbidity")
S38	(MH "Noncommunicable Diseases")
S37	S12 OR S13 OR S14 OR S15 OR S16 OR S17 OR S18 OR S19 OR S20 OR S21 OR S22 OR S23 OR S24 OR S25 OR S26 OR S27 OR S28 OR S29 OR S30 OR S31 OR S32 OR S33 OR S34 OR S35 OR S36
S36	TI dancing OR AB dancing OR TW dancing
S35	(MH "Dancing+")
S34	TI gymnastics OR AB gymnastics OR TW gymnastics
S33	TI swimming OR AB swimming OR TW swimming
S32	TI cycling OR AB cycling OR TW cycling
S31	TI bicycling OR AB bicycling OR TW bicycling
S30	TI jogging OR AB jogging OR TW jogging
S29	TI running OR AB running OR TW running
S28	(MH "Running+")
S27	TI walking OR AB walking OR TW walking
S26	(MH "Walking+")
S25	(MH "Motor Activity+")
S24	(MH "Physical Therapy Practice, Research-Based")
S23	(MH "Physical Therapy+")
S22	(MH "Locomotion+")
S21	TI exercise therapy OR AB exercise therapy OR TW exercise therapy
S20	(MH "Therapeutic Exercise+")
S19	TI exercis* OR AB exercis* OR TW exercis*

S18	(MH "Exercise+")
S17	TI aerobics OR AB aerobics OR TW aerobics
S16	(MH "Aerobic Exercises+")
S15	TI fitness OR AB fitness OR TW fitness
S14	(MH "Physical Fitness+")
S13	(MH "Sports+")
S12	(MH "Physical Activity")
S11	S1 OR S2 OR S3 OR S4 OR S5 OR S6 OR S7 OR S8 OR S9 OR S10
S10	(TI cell* OR AB cell* OR TW cell* or TI smart* OR AB smart* OR TW smart* or TI mobile OR AB mobile OR TW mobile or TI android OR AB android OR TW android or TI internet OR AB internet OR TW internet or TI web OR AB web OR TW web) n3 (TI comput* OR AB comput* OR TW comput* or TI device OR AB device OR TW device or TI app* OR AB app* OR TW app* or TI phone OR AB phone OR TW phone)
S9	(TI arm band OR AB arm band OR TW arm band OR TI wrist band OR AB wrist band OR TW wrist band OR TI heart rate OR AB heart rate OR TW heart rate OR TI heart-rate OR AB heart-rate OR TW heart-rate or TI heart rhythm OR AB heart rhythm OR TW heart rhythm or TI heart-rhythm OR AB heart-rhythm OR TW heart-rhythm or TI step OR AB step OR TW step or TI measuring OR AB measuring OR TW measuring or TI electronic* OR AB electronic* OR TW electronic* or TI mechanic* OR AB mechanic* OR TW mechanic* or TI portable OR AB portable OR TW portable or TI wearable OR AB wearable OR TW wearable or TI motion OR AB motion OR TW motion or TI physical activity OR AB physical activity OR TW physical activity or TI fitness OR AB fitness OR TW fitness or TI activity OR AB activity OR TW activity) n3 (TI track* OR AB track* OR TW track* or TI device* OR AB device* OR TW device* or TI sensor* OR AB sensor* OR TW sensor* or TI detector* OR AB detector* OR TW detector* or TI monitor* OR AB monitor* OR TW monitor* or TI count* OR AB count* OR TW count*)
S8	TI objective measurement* OR AB objective measurement* OR TW objective measurement*
S7	TI direct measurement* OR AB direct measurement* OR TW direct measurement*
S6	TI direct observation* OR AB direct observation* OR TW direct observation*
S5	TI multi-sensor* OR AB multi-sensor* OR TW multi-sensor*
S4	TI multisensor* OR AB multisensor* OR TW multisensor*
S3	TI motion sensor* OR AB motion sensor* OR TW motion sensor*
S2	TI pedomet* OR AB pedomet* OR TW pedomet*
S1	TI acceleromet* or AB acceleromet* OR TW acceleromet*
