# Supplementary material for: Objectively measured physical activity levels and adherence to physical activity guidelines in people with multimorbidity—A systematic review and meta-analysis
Source: PLoS One. 2022 Oct 12;17(10):e0274846. doi: 10.1371/journal.pone.0274846 (PMC9555650; doi:10.1371/journal.pone.0274846)
Supplement: S5 File — (PDF) [file pone.0274846.s005.pdf]

S5 Search strategy CENTRAL

#1 accelerometet*:ti,ab,kw

#2 pedomet*:ti,ab,kw

#3 motion sensor*:ti,ab,kw

#4 multisensor*:ti,ab,kw

#5 multi-sensor*:ti,ab,kw

#6 direct observation*:ti,ab,kw

#7 direct measurement*:ti,ab,kw

#8 objective measurement*:ti,ab,kw

#9 (arm band or wrist band or heart rate or heart-rate or heart rhythm or heart-rhythm or step or measuring or electronic* or mechanic* or portable or wearable or motion or physical activity or fitness or activity) NEAR/3 (track* or device* or sensor* or detector* or monitor* or count*):ti,ab,kw

#10 (cell* or smart* or mobile or android or internet or web) NEAR/3 (comput* or device or app* or phone):ti,ab,kw

#11 #1 or #2 or #3 or #4 or #5 or #6 or #7 or #8 or #9 or #10

#12 Physical activit*:ti,ab,kw

#13 MeSH descriptor: [Sports] explode all trees

#14 Sport*:ti,ab,kw

#15 MeSH descriptor: [Physical Fitness] explode all trees

#16 "fitness":ti,ab,kw

#17 aerobics:ti,ab,kw

#18 MeSH descriptor: [Exercise] explode all trees

#19 exercis*:ti,ab,kw

#20 MeSH descriptor: [Exercise Therapy] explode all trees

#21 exercise therap*:ti,ab,kw

#22 MeSH descriptor: [Locomotion] explode all trees

#23 MeSH descriptor: [Physical Therapy Modalities] explode all trees

#24 physiotherapy:ti,ab,kw

#25 physical therapy:ti,ab,kw

#26 MeSH descriptor: [Motor Activity] explode all trees

#27 MeSH descriptor: [Walking] explode all trees

#28 walking:ti,ab,kw

#29 MeSH descriptor: [Running] explode all trees

#30 running:ti,ab,kw

#31 MeSH descriptor: [Jogging] explode all trees

#32 jogging:ti,ab,kw

#33 MeSH descriptor: [Bicycling] explode all trees

#34 bicycling:ti,ab,kw

#35 "cycling":ti,ab,kw

#36 MeSH descriptor: [Swimming] explode all trees

#37 swim*:ti,ab,kw

#38 MeSH descriptor: [Gymnastics] explode all trees

#39 gymnastic*:ti,ab,kw

#40 MeSH descriptor: [Dancing] explode all trees

#41 dancing:ti,ab,kw

#42 #12 or #13 or #14 or #15 or #16 or #16 or #17 or #18 or #19 or #20 or #21 or #22 or #23 or #24 or #25 or #26 or #27 or #28 or #29 or #30 or #31 or #32 or #33 or #34 or #35 or #36 or #37 or #39 or #40 or #41

#43 MeSH descriptor: [Multimorbidity] explode all trees

#44 multimorbid*:ti,ab,kw

#45 multi-morbid*:ti,ab,kw

#46 MeSH descriptor: [Multiple Chronic Conditions] explode all trees

#47 MeSH descriptor: [Comorbidity] explode all trees

#48 comorbid*:ti,ab,kw

#49 co-morbid*:ti,ab,kw

#50 MeSH descriptor: [Noncommunicable Diseases] explode all trees

#51 (concurrent or simultaneous or dual or multi or multiple or pluri or poly or chronic* or coexist* or co-exist* or co-occur* or cooccur*) NEAR/3 (condition* or disease* or illness* or disorder* or morbidit* or patholog* or diagnos* or syndrome* or health problem*):ti,ab,kw

#52 #43 or #44 or #45 or #46 or #47 or #48 or #49 or #50 or #51

#53 MeSH descriptor: [Myocardial Ischemia] explode all trees

#54 myocardial ischemia:ti,ab,kw

#55 MeSH descriptor: [Coronary Artery Disease] explode all trees

#56 coronary artery disease:ti,ab,kw

#57 MeSH descriptor: [Coronary Disease] explode all trees

#58 coronary disease:ti,ab,kw

#59 MeSH descriptor: [Myocardial Infarction] explode all trees

#60 myocardial infarction:ti,ab,kw

#61 MeSH descriptor: [Angina Pectoris] explode all trees

#62 angina pectoris:ti,ab,kw

#63 MeSH descriptor: [Heart Failure] explode all trees

#64 heart failure:ti,ab,kw

#65 "HFNEF":ti,ab,kw

#66 "HFPEF":ti,ab,kw

#67 "HFREF":ti,ab,kw

#68 "HF NEF":ti,ab,kw

#69 "HF PEF":ti,ab,kw

#70 "HF REF":ti,ab,kw

#71 MeSH descriptor: [Heart Diseases] explode all trees

#72 heart disease*:ti,ab,kw

#73 MeSH descriptor: [Coronary Artery Bypass] explode all trees

#74 coronary artery bypass:ti,ab,kw

#75 #53 or #54 or #55 or #56 or #57 or #58 or #59 or #60 or #61 or #62 or #63 or #64 or #65 or #66 or #67 or #68 or #69 or #70 or #71 or #72 or #73 or #74

#76 MeSH descriptor: [Pulmonary Disease, Chronic Obstructive] explode all trees

#77 COPD:ti,ab,kw

#78 MeSH descriptor: [Pulmonary Emphysema] explode all trees

#79 pulmonary emphysema:ti,ab,kw

#80 coad:ti,ab,kw

#81 MeSH descriptor: [Bronchitis, Chronic] explode all trees

#82 chronic bronchitis:ti,ab,kw

#83 chronic obstructive lung disease:ti,ab,kw

#84 #76 or #77 or #78 or #79 or #80 or #81 or #82 or #83

#85 MeSH descriptor: [Hypertension] explode all trees

#86 hypertens*:ti,ab,kw

#87 high blood pressure:ti,ab,kw

#88 MeSH descriptor: [Blood Pressure] explode all trees

#89 #85 or #86 or #87 or #88

#90 MeSH descriptor: [Depression] explode all trees

#91 depression:ti,ab,kw

#92 MeSH descriptor: [Dysthymic Disorder] explode all trees

#93	dysthymi*:ti,ab,kw
#94	(dysthymic or affect*) NEAR/2 (disorder* or symptom*):ti,ab,kw
#95	#90 or #91 or #92 or #93 or #94
#96	MeSH descriptor: [Anxiety] explode all trees
#97	anxiety:ti,ab,kw
#98	MeSH descriptor: [Phobia, Social] explode all trees
#99	anxiety disorder*:ti,ab,kw
#100	#96 or #97 or #98 or #99
#101	MeSH descriptor: [Diabetes Mellitus] explode all trees
#102	diabetes mellitus:ti,ab,kw
#103	MeSH descriptor: [Diabetes Mellitus, Type 2] explode all trees
#104	Type 2 diab*:ti,ab,kw
#105	Type II diab*:ti,ab,kw
#106	Non-Insulin-Dependent Diabetes Mellitus:ti,ab,kw
#107	NIDDM:ti,ab,kw
#108	impaired glucose toleranc*:ti,ab,kw
#109	MeSH descriptor: [Glucose Intolerance] explode all trees
#110	blood glucose:ti,ab,kw
#111	#101 or #102 or #103 or #104 or #105 or #106 or #107 or #108 or #109 or #110
#112	MeSH descriptor: [Osteoarthritis] explode all trees
#113	osteoarthrit*:ti,ab,kw
#114	osteoarthros*:ti,ab,kw
#115	#112 or #113 or #114
#116	MeSH descriptor: [Spinal Stenosis] explode all trees
#117	spin* NEAR/5 stenos*:ti,ab,kw
#118	lumbar NEAR/5 stenos*:ti,ab,kw
#119	neuro* NEAR/2 claud*:ti,ab,kw
#120	lumbar radicular pain:ti,ab,kw
#121	MeSH descriptor: [Cauda Equina] explode all trees
#122	Cauda Equina:ti,ab,kw
#123	MeSH descriptor: [Spinal Osteophytosis] explode all trees
#124	Spinal Osteophytosis:ti,ab,kw
#125	MeSH descriptor: [Spondylosis] explode all trees

#126 Spondylos*:ti,ab,kw

#127 MeSH descriptor: [Spondylolisthesis] explode all trees

#128 Spondylolisthesis:ti,ab,kw

#129 MeSH descriptor: [Low Back Pain] explode all trees

#130 low* NEAR/5 back NEAR/5 pain:ti,ab,kw

#131 #116 or #117 or #118 or #119 or #120 or #121 or #122 or #123 or #124 or #125 or #126 or #127 or #128 or #129 or #130

#132 #75 and (#84 or #89 or #95 or #100 or #111 or #115 or #131)

#133 #84 and (#89 or #95 or #100 or #111 or #115 or #131)

#134 #89 and (#95 or #100 or #111 or #115 or #131)

#135 #95 and (#100 or #111 or #115 or #131)

#136 #100 and (#111 or #115 or #131)

#137 #111 and (#115 or #131)

#138 #115 or #131

#139 #11 and #42 and (#52 or #132 or #133 or #134 or #135 or #136 or #137 or #138)

#140 animals not humans.sh

#141 #139 not #140
