# Supplementary material for: Objectively measured physical activity levels and adherence to physical activity guidelines in people with multimorbidity—A systematic review and meta-analysis
Source: PLoS One. 2022 Oct 12;17(10):e0274846. doi: 10.1371/journal.pone.0274846 (PMC9555650; doi:10.1371/journal.pone.0274846)
Supplement: S6 File — (PDF) [file pone.0274846.s006.pdf]

S6 Search strategy for grey literature

Website name	Search strategy
Clinicaltrials.gov	*'Condition/disease' field*<br><br>multimorbidity OR multi-morbidity OR comorbidity OR chronic disease OR chronic illness OR chronic condition<br><br>*'Other terms' field*<br>accelerometer OR pedometer OR motion sensor OR multisensor OR multi-sensor OR direct observation OR direct measurement OR objective measurement OR monitor OR device OR activity tracker OR motion OR device OR measure<br><br>*'Limits'*<br>Completed<br>With results<br>Adults (18-64 years)<br>Older adults (65+)
Opengrey.eu	1: multimorbidity<br><br>2: (multimorbidity OR multi-morbidity OR comorbidity OR chronic disease OR chronic illness OR chronic condition) AND (accelerometer OR pedometer OR motion sensor OR multisensor OR multi-sensor OR direct observation OR direct measurement OR objective measurement OR monitor OR device OR activity tracker OR motion OR device OR measure)<br><br>3: accelerometer OR pedometer
Google.com	1: multimorbidity and objectively measured physical activity<br><br>All the results from the search were reviewed for relevance by one reviewer (LBJ)
