# Supplementary material for: Objectively measured physical activity levels and adherence to physical activity guidelines in people with multimorbidity—A systematic review and meta-analysis
Source: PLoS One. 2022 Oct 12;17(10):e0274846. doi: 10.1371/journal.pone.0274846 (PMC9555650; doi:10.1371/journal.pone.0274846)
Supplement: S7 File — (PDF) [file pone.0274846.s007.pdf]

S7 Quality Assessment Tool

This quality assessment tool was developed specifically for use in the quality assessment of studies included in the systematic review and meta-analysis ‘Systematic review and meta-analysis of objectively measured physical activity level and adherence to physical activity guidelines in people with multimorbidity’ - (PROSPERO reg. number - CRD42020172456).

The components of the quality assessment tool were developed with inspiration from the Quality Assessment Tool for Quantitative Studies developed by the Effective Public Health Practice Project (https://www.ephpp.ca/quality-assessment-tool-for-quantitative-studies/).

Each section of the quality assessment tool has a small ‘dictionary’ describing items in the tool thereby assisting raters to score study quality. When making judgements about each component, raters should only form their opinion based upon information contained in the study and not inferences about what the authors intended.

Step 1. Rate the study independently using the checklist

Rating and comments must be made in the table at the end of this appendix (see table, step 2).

SELECTION BIAS

Q1 How representative was the study participants of the multimorbid target population?

1. Very representative
2. Somewhat representative
3. Not representative

Q2 Percentage of eligible study participants that participated

1. 80 - 100%
2. 60 – 79%
3. Less than 60%/not reported

Q3 Percentage of study participants that contributed with data on physical activity

1. 80 - 100%
2. 60 – 79%
3. Less than 60%/not reported

Strong	Moderate	Weak
Q1, Q2 and Q3 are all answered with 1	Any other option	Either Q1, Q2 or Q3 is answered with 3

Dictionary for rating of selection bias

A ‘multimorbid target population’ are to be defined as a population of people that are multimorbid based on the two (or more) chronic conditions that made them eligible for inclusion in this systematic review – e.g. heart failure and depression. When making judgement of representativeness of the study participants, a rater should carefully consider all aspects that could affect the representativeness: if participants were enrolled consecutively? If all patients or only selected groups of patients were invited to participate? If studies used exclusion criteria that obviously excluded specific parts of a target population e.g. people with heart failure and an ejection fraction <40%.

BLINDING

Q1 Was the study participants blinded to the data on physical activity collected with the objective measurement methods?

1. Yes
2. No
3. Not reported

Rating

Strong	Weak
Q1 is answered with 1	Any other option

Dictionary for rating of blinding

To obtain a rating of strong, the study participants should be blinded to the collection of physical activity data to protect against reporting bias. With accelerometers, data are rarely accessible to the participant but with consumer-based activity-trackers such as e.g. Garmin Vivofit or pedometers, data can be accessible to the user/participant. Attention: Blinding cannot be rated moderate.

DATA COLLECTION METHODS

Q1 Has the measurement tool used to collect physical activity data shown to be valid?

1. Yes
2. No
3. Validity of the tool is not reported.

Q2 Has the measurement tool used to collect physical activity data shown to be reliable?

1. Yes
2. No
3. Reliability of the tool is not reported.

Rating

Strong	Moderate	Weak
Both Q1 and Q2 are answered with 1	Either Q1 or Q2 are answered with 1	Any other option

Dictionary for rating of data collection methods

Tools for primary outcome measures must be described as reliable and valid. If ‘face’ validity or ‘content’ validity has been demonstrated, this is acceptable. Reliability and validity could be reported in a separate study and some measurement tools have known reliability and validity, but a study will be downgraded if validity and reliability are not reported in the study.

Global rating of the study

Step 2. Resolve discrepancies and establish consensus on rating

**Selection Bias**		
	**Comments reviewer 1**	**Comments reviewer 2**
Q1	Rating:<br>Comments:	Rating:<br>Comments:
Q2	Rating:<br>Comments:	Rating:<br>Comments:
Q3	Rating:<br>Comments:	Rating:<br>Comments:
	Agreed rating: Q1=, Q2=, Q3=	
**Blinding**		
	**Comments reviewer 1**	**Comments reviewer 2**
Q1	Rating:<br>Comments:	Rating:<br>Comments:

	Agreed rating: Q1=	
**Data collection methods**		
	**Comments reviewer 1**	**Comments reviewer 2**
Q1	Rating:<br>Comments:	Rating:<br>Comments:
Q2	Rating:<br>Comments:	Rating:<br>Comments:
	Agreed rating: Q1=, Q2=	

Step 3. Make final decision of rating

**Strong**	**Moderate**	**Weak**
Only strong ratings	Only strong and moderate ratings	One or more weak rating
**High quality/low risk of bias**	**Moderate quality/moderate risk of bias**	**Low quality/high risk of bias**

Final decision
