# Supplementary material for: Objectively measured physical activity levels and adherence to physical activity guidelines in people with multimorbidity—A systematic review and meta-analysis
Source: PLoS One. 2022 Oct 12;17(10):e0274846. doi: 10.1371/journal.pone.0274846 (PMC9555650; doi:10.1371/journal.pone.0274846)
Supplement: S8 File — (PDF) [file pone.0274846.s008.pdf]

S8 Studies excluded in full text screening (n=345)

1.
Abel B., Bongartz M., Eckert T., Ullrich P., Beurskens R., Mellone S., et al. Will We Do If We Can? Habitual Qualitative and Quantitative Physical Activity in Multi-Morbid, Older Persons with Cognitive Impairment. Sensors (Basel, Switzerland) [Internet]. 2020;20(24). Available from: https://res.mdpi.com/d_attachment/sensors/sensors-20-07208/article_deploy/sensors-20-07208-v2.pdf
2.
ACTRN12615000015549. Prescribing the maximum tolerated dose of walking for people with severe knee osteoarthritis: a Phase II, Randomised Controlled Trial. http://www.who.int/trialsearch/Trial2.aspx?TrialID=ACTRN12615000015549. 2015;
3.
ACTRN12617001404314. Accelerometer-based facilitated walking program in addition to usual care for the management of patients with low back pain at medium or high risk of chronicity: a randomised controlled trial. http://www.who.int/trialsearch/Trial2.aspx?TrialID=ACTRN12617001404314. 2017;
4.
Adams DJ, Remick RA, Davis JC, Vazirian S, Khan KM. Exercise as medicine-the use of group medical visits to promote physical activity and treat chronic moderate depression: a preliminary 14-week pre-post study. BMJ open sport & exercise medicine. 2015;1(1):e000036.
5.
Agarwal V., Tetenta S., Bautista J., ZuWallack R., Lahiri B. Longitudinal changes in directly measured physical activity in patients with chronic obstructive pulmonary disease. Journal of Cardiopulmonary Rehabilitation and Prevention. 2012;32(5):292–5.
6.
Aguilar BAS, Tebar WR, Silva SCB, Gomes LQ, Damato TMM, Mota J, et al. Leisure-time exercise is associated with lower depressive symptoms in community dwelling adults. European journal of sport science. 2021;(101146739):1–29.
7.
Ahamed Y., Egerton T., Hunt M.A., Keefe F.J., Bryant C., Jull G., et al. Psychological factors associated with daily step count in knee osteoarthritis. Osteoarthritis and Cartilage. 2012;20(SUPPL. 1):S191–2.
8.
Aitken D, Wu F, Jones G, Balogun SA, Scott D, Winzenberg T. Linear and Nonlinear Associations Between Physical Activity, Body Composition, and Multimorbidity Over 10 Years Among Community-Dwelling Older Adults. J Gerontol A Biol Sci Med Sci. 2021;76(11):2015–20.
9.
Alahmari AD, Patel AR, Kowlessar BS, Mackay AJ, Singh R, Wedzicha JA, et al. Daily activity during stability and exacerbation of chronic obstructive pulmonary disease. BMC Pulmonary Medicine. 2014;14(1):98–98.
10.
Alghafri T.S., Alharthi S.M., Al-Farsi Y., Alrawahi A.H., Bannerman E., Craigie A.M., et al. 'MOVEdiabetes': A cluster randomized controlled trial to increase physical activity in adults with type 2 diabetes in primary health in Oman. BMJ Open Diabetes Research and Care. 2018;6(1):e000605.
11.

Aljaloud K. Habitual physical activity assessment using objective measuring devices : observations in lean and obese adults and children [Internet]. 2010 [cited 2021 Mar 1]. Available from: http://hdl.handle.net/10068/974525
12.
Allen K., Hall K., Lindquist J.H., Taylor S., Coffman C. The value of adjusting for physical activity when measuring osteoarthritis-related pain. Arthritis and Rheumatology. 2016;68(Supplement 10):407–9.
13.
Alonso-Dominguez R., Patino-Alonso M.C., Sanchez-Aguadero N., Garcia-Ortiz L., Recio-Rodriguez J.I., Gomez-Marcos M.A. Effect of a multifactorial intervention on the increase in physical activity in subjects with type 2 diabetes mellitus: a randomized clinical trial (EMID Study). European Journal of Cardiovascular Nursing. 2019;18(5):399–409.
14.
Alosco ML, Miller L, Cohen R, Colbert LH, Waechter D, Rosneck J, et al. Depression Is Associated With Reduced Physical Activity in Persons With Heart Failure. Health Psychology. 2012;31(6):754–62.
15.
Alzahrani H, Mackey M, Stamatakis E, Shirley D. Wearables-based walking program in addition to usual physiotherapy care for the management of patients with low back pain at medium or high risk of chronicity: A pilot randomized controlled trial. PLoS ONE. 2021;16(8):e0256459.
16.
Amorim P.B., Stelmach R., Carvalho C.R., Fernandes F.L., Carvalho-Pinto R.M., Cukier A. Barriers associated with reduced physical activity in COPD patients. Jornal brasileiro de pneumologia : publicacao oficial da Sociedade Brasileira de Pneumologia e Tisilogia. 2014;40(5):504–12.
17.
Anderson D, Harris K, Landers J, Emery C. Lower anxiety associated with greater physical activity in a pedometer-based intervention among cardiac patients. Psychosomatic medicine. 2015;77(3):A17-A18.
18.
Andreae C., Arestedt K., Evangelista L., Stromberg A. The relationship between physical activity and appetite in patients with heart failure: A prospective observational study. European Journal of Cardiovascular Nursing. 2019;18(5):410–7.
19.
Aoyagi Y., Park S., Cho S., Shephard R.J. Objectively measured habitual physical activity and sleep-related phenomena in 1645 people aged 1-91years: The Nakanojo Community Study. Preventive Medicine Reports. 2018;11((Aoyagi, Park, Cho) Exercise Sciences Research Group, Tokyo Metropolitan Institute of Gerontology, Itabashi, Tokyo, Japan):180–6.
20.
Araujo M, Baldi B, Freitas C, Albuquerque A, Marques da Silva C, Kairalla R, et al. Pulmonary rehabilitation in lymphangioleiomyomatosis: a controlled clinical trial. The european respiratory journal. 2016;47(5):1452-1460.
21.
Arbeeva L, Cleveland RJ, Golightly YM, Hales DP, Allen KD, Beauchamp J. Daily patterns of sedentary physical activity associated with self-reported outcomes among individuals with osteoarthritis. Osteoarthritis Cartilage. 2021;29(Supplement 1):S255–6.
22.

Axelsson T.G., Lindholm B., Carrero J.J., Heimburger O., Stenvinkel P., Qureshi A.R. Association between physical activity measured by accelerometry and inflammation, co-morbidity, and nutritional status in peritoneal dialysis patients. Nephrology Dialysis Transplantation [Internet]. 2012;27(SUPPL. 2). Available from: https://watermark.silverchair.com/gfs243.pdf?token=AQECAHi208BE49Ooan9kkhW_Ercy7Dm3ZL_9Cf3qfKAc485ysgAAArcwggKzBgkqhkiG9w0BBwagggKkMIICoAIBADCCApkGCSqGSIb3DQEHATAeBglghkgBZQMEAS4wEQQMFtP4I2g4CnOffuNhAgEQgIICavdLCa9KTWKVxWRSLaYM8Wr5TUNAO6xDYJd18C-rxL-MHXT0
23.
Ballin M., Nordstrom P., Niklasson J., Alamaki A., Condell J., Tedesco S., et al. Daily step count and incident diabetes in community-dwelling 70-year-olds: a prospective cohort study. BMC public health. 2020;20(1):1830.
24.
Ballin M., Nordstrom P., Nordstrom A. Associations of Light, Moderate to Vigorous, and Total Physical Activity With the Prevalence of Metabolic Syndrome in 4,652 Community-Dwelling 70-Year-Olds: A Population-Based Cross-Sectional Study. Journal of aging and physical activity. 2021;1–9.
25.
Barker J., Smith Byrne K., Doherty A., Foster C., Rahimi K., Ramakrishnan R., et al. Corrigendum: Physical activity of UK adults with chronic disease: Cross sectional analysis of accelerometer measured physical activity in 96 706 UK Biobank participants (International Journal of Epidemiology (2019) DOI: 10.1093/ije/dyy294). International Journal of Epidemiology. 2019;48(4):1386.
26.
Barker J, Byrne KS, Doherty A, Foster C, Rahimi K, Ramakrishnan R, et al. Physical activity of UK adults with chronic disease: cross-sectional analysis of accelerometer-measured physical activity in 96 706 UK Biobank participants. International Journal of Epidemiology. 2019;48(4):1167–74.
27.
Barnes A, Newby C, Chaplin E, Houchen-Wolloff L, Singh S. Purposeful physical activity in COPD patients comparing standard and web based pulmonary rehabilitation. European respiratory journal. 2016;48.
28.
Barrett T.M., Liebert M.A., Schrock J.M., Cepon-Robins T.J., Mathur A., Agarwal H., et al. Physical function and activity among older adults in Jodhpur, India. Annals of human biology. 2016;43(5):488–91.
29.
Bell J.A., Hamer M., Van Hees V.T., Singh-Manoux A., Kivimaki M., Sabia S. Healthy obesity and objective physical activity. American Journal of Clinical Nutrition. 2015;102(2):268–75.
30.
Bellettiere J., LaMonte M.J., Rillamas-Sun E., Kerr J., Evenson K.R., Lee I.-M., et al. Sedentary behavior increases risk for cardiovascular disease in older women: The objective physical activity and cardiovascular health (opach) study. Circulation. 2018;137(Supplement 1).
31.
Belza B, Steele BG, Hunziker J, Lakshminaryan S, Holt L, Buchner DM. Correlates of physical activity in chronic obstructive pulmonary disease. Nursing Research. 2001;50(4):195–202.
32.

Bernard P., Hains-Monfette G., Atoui S., Moullec G. Daily Objective Physical Activity and Sedentary Time in Adults with COPD Using Spirometry Data from Canadian Measures Health Survey. Canadian Respiratory Journal. 2018;2018((Bernard, Hains-Monfette, Atoui) Department of Physical Activity Sciences, Universite du Quebec A Montreal, Montreal, QC, Canada):9107435.
33.
Bernocchi P, Vitacca M, La Rovere MT, Volterrani M, Galli T, Baratti D, et al. Home-based telerehabilitation in older patients with chronic obstructive pulmonary disease and heart failure: a randomised controlled trial. Age Ageing. 2018 Jan 1;47(1):82–8.
34.
Bielemann R.M., Silveira M.P.T., Lutz B.H., Miranda V.I.A., Gonzalez M.C., Brage S., et al. Objectively Measured Physical Activity and Polypharmacy Among Brazilian Community-Dwelling Older Adults. Journal of physical activity & health. 2020;1–7.
35.
Bielemann RM, Silveira MPT, Lutz BH, Miranda VIA, Gonzalez MC, Brage S, et al. Objectively Measured Physical Activity and Polypharmacy Among Brazilian Community-Dwelling Older Adults. J Phys Act Health. 2020 May 29;1–7.
36.
Blom E.E., Aadland E., Skrove G.K., Solbraa A.K., Oldervoll L.M. Health-related quality of life and physical activity level after a behavior change program at Norwegian healthy life centers: a 15-month follow-up. Quality of Life Research. 2020;29(11):3031–41.
37.
Blumenthal JA, Babyak MA, O'Connor C, Keteyian S, Landzberg J, Howlett J, et al. Effects of Exercise Training on Depressive Symptoms in Patients with Chronic Heart Failure: The HF-ACTION Randomized Trial. JAMA. 2012 Aug 1;308(5):465–74.
38.
Britto A, Kumar S, Hazari A, Jadhav R, Babu AS, Bhat V, et al. Physical Activity Measurement Usingan Accelerometer among Indians with Type 2 Diabetes Mellitus: A Cross-Sectional Study. Critical Reviews in Physical & Rehabilitation Medicine. 2018;30(2):141–50.
39.
Broers E, Gavidia G, Wetzels M, Ribas V, Ayoola I, Piera-Jimenez J, et al. Usefulness of a Lifestyle Intervention in Patients With Cardiovascular Disease. American journal of cardiology. 2020;125(3):370-375.
40.
Brown S.J., Vileikyte L., Boulton A.J.M., Reeves N.D. Physical and psychological determinants of fall risk in patients with diabetic neuropathy: A prospective investigation. Diabetologia. 2018;61(Supplement 1):S466.
41.
Buriakovska O., Isayeva G. Sleep Quality And Plasma Lipids In Patients With Hypertension And Diabetes Mellitus. Atherosclerosis. 2019;287((Buriakovska, Isayeva) The Government Institution 'National Institute of Therapy named by L.T. Malaya of National Ukrainian Academy of Medical Science', Department of Chronic Non-communicable Disease Prevention, Kharkiv, Ukraine):e186.
42.
Burrage D.R., Wells C.E., Hull J.H.K., Baker E.H. Physical activity and cardiovascular risk in people with stable COPD. American Journal of Respiratory and Critical Care Medicine. 2014;189(MeetingAbstracts).
43.

Byun W, Ozemek C, Riggin K, Strath S, Kaminsky L. Correlates of objectively measured physical activity in cardiac patients. Cardiovascular Diagnosis and Therapy. 2014 Oct;4(5):40610–40410.
44.
Camcioglu B, Bosnak Guclu M, Dundar Z, Aydogdu Tacoy G, Cengel A. Physical activity, functional exercise capacity, respiratory and peripheral muscle strength, depression and fatigue in patients with pulmonary arterial hypertension. Fizyoterapi rehabilitasyon. 2018;29(1):S35-.
45.
Camcioglu B, Guclu M, Dundar Z, Tacoy G, Cengel A. Physical activity, functional exercise capacity, respiratory and peripheral muscle strength, depression and fatigue in patients with pulmonary arterial hypertension. Anatolian journal of cardiology. 2017;18:98-.
46.
Camhi S.M., Sisson S.B., Johnson W.D., Katzmarzyk P.T., Tudor-Locke C. Accelerometer-determined moderate intensity lifestyle activity and cardiometabolic health. Preventive Medicine. 2011;52(5):358–60.
47.
Canavan J.I., Kon S.S.C., Nolan C.M., Jones S.E., Polkey M.I., Man W.D.-C. Physical activity levels according to gold grouping in patients with COPD. Thorax. 2013;68(SUPPL. 3):A91.
48.
Cassidy S., Fuller H., Chau J., Catt M., Bauman A., Trenell M.I. Accelerometer-derived physical activity in those with cardio-metabolic disease compared to healthy adults: a UK Biobank study of 52,556 participants. Acta Diabetologica. 2018;55(9):975–9.
49.
Caulfield B., Kaljo I., Donnelly S. Use of a consumer market activity monitoring and feedback device improves exercise capacity and activity levels in COPD. Conference proceedings : . Annual International Conference of the IEEE Engineering in Medicine and Biology Society IEEE Engineering in Medicine and Biology Society Annual Conference. 2014;2014:1765–8.
50.
Chan C.B., Ryan D.A.J., Tudor-Locke C. Health benefits of a pedometer-based physical activity intervention in sedentary workers. Preventive Medicine. 2004;39(6):1215–22.
51.
Chan C.B., Spangler E., Valcour J., Tudor-Locke C. Cross-sectional relationship of pedometer-determined ambulatory activity to indicators of health. Obesity research. 2003;11(12):1563–70.
52.
Chang A., Song J., Lee J., Chang R., Semanik P., Dunlop D. Impact of eliminating the bouted minutes requirement in the new 2018 physical activity guidelines for Americans on gender disparity in guideline attainment for persons with or at high risk of knee osteoarthritis. Arthritis and Rheumatology. 2019;71(Supplement 10):3661–3.
53.
Chang A.H., Song J., Lee J., Chang R.W., Semanik P.A., Dunlop D.D. Proportion and associated factors of meeting the 2018 Physical Activity Guidelines for Americans in adults with or at risk for knee osteoarthritis. Osteoarthritis and Cartilage. 2020;28(6):774–81.
54.
Chapman J.J., Fraser S.J., Brown W.J., Burton N.W. The feasibility and acceptability of questionnaires and accelerometry for measuring physical activity and sedentary behaviour in adults with mental illness. Journal of Mental Health. 2015;24(5):299–304.
55.

Chudasama YV, Khunti KK, Zaccardi F, Rowlands AV, Yates T, Gillies CL, et al. Physical activity, multimorbidity, and life expectancy: a UK Biobank longitudinal study. BMC Medicine. 2019;17(1):1–13.
56.
Chudasama YV, Khunti KK, Zaccardi F, Rowlands AV, Yates T, Gillies CL, et al. Physical activity, multimorbidity, and life expectancy: a UK Biobank longitudinal study. BMC Medicine. 2019 Jun 12;17(1):108.
57.
Cichosz S.L., Fleischer J., Hoeyem P., Laugesen E., Poulsen P.L., Christiansen J.S., et al. Objective measurements of activity patterns in people with newly diagnosed Type 2 diabetes demonstrate a sedentary lifestyle. Diabetic Medicine. 2013;30(9):1063–6.
58.
Clarenbach C., Sievi N., Senn O., Brack T., Brutsche M., Frey M., et al. The impact of comorbidities on physical activity in COPD. Respiration. 2014;87(6):548.
59.
Clarenbach C.F., Sievi N.A., Brack T., Brutsche M., Frey M., Irani S., et al. Longitudinal assessment of physical activity in patients with COPD. Kardiovaskulare Medizin. 2016;19(5 Supplement 26):17S.
60.
Clarenbach C.F., Sievi N.A., Haile S.R., Brack T., Brutsche M.H., Frey M., et al. Determinants of annual change in physical activity in COPD. Respirology. 2017;22(6):1133–9.
61.
Clarke CL, Sniehotta FF, Vadiveloo T, Argo IS, Donnan PT, McMurdo MET, et al. Factors associated with change in objectively measured physical activity in older people - data from the physical activity cohort Scotland study. BMC Geriatrics. 2017;17:1–9.
62.
Colpitts B.H., Smith S., Bouchard D.R., Boudreau J., Senechal M. Are physical activity and sedentary behavior patterns contributing to diabetes and metabolic syndrome simultaneously? Translational Sports Medicine. 2020;((Colpitts, Smith, Bouchard, Senechal) Cardiometabolic Exercise and Lifestyle Laboratory, University of New Brunswick, Fredericton, NB, Canada).
63.
Cooke A.B., Daskalopoulou S.S., Dasgupta K. Step counts and sedentary time in type 2 diabetes and hypertension: Seasonal variations. Obesity Reviews. 2016;17(SUPPL. 2):84–5.
64.
Cooke A, Daskalopoulou S, Dasgupta K. The impact of accelerometer wear location on the relationship between step counts and arterial stiffness in adults treated for hypertension and diabetes. Journal of science and medicine in sport. 2018;21(4):398-403.
65.
Cooke AB, Daskalopoulou SS, Dasgupta K. 149 - Seasonal Variations in Step Counts, Physical Activity Intensity and Sedentary Behaviour in Type 2 Diabetes and Hypertension. Canadian Journal of Diabetes. 2016;40:S55–S55.
66.
Cooke AB, Rahme E, Defo AK, Chan D, Daskalopoulou SS, Dasgupta K. A trajectory analysis of daily step counts during a physician-delivered intervention. Journal of Science & Medicine in Sport. 2020;23(10):962–7.
67.

Cooper A, Brage S, Ekelund U, Wareham N, Griffin S, Simmons R. Association between objectively assessed sedentary time and physical activity with metabolic risk factors among people with recently diagnosed type 2 diabetes. Diabetologia. 2014;57(1):73-82.
68.
Cordova-Rivera L., Gibson P.G., Gardiner P.A., McDonald V.M. Physical activity associates with disease characteristics of severe asthma, bronchiectasis and COPD. Respirology. 2019;24(4):352–60.
69.
Crook S., Busching G., Keusch S., Wieser S., Turk A., Frey M., et al. The association between daily exacerbation symptoms and physical activity in patients with chronic obstructive pulmonary disease. International Journal of COPD. 2018;13((Crook, Puhan, Frei) Department of Epidemiology, Epidemiology, Biostatistics and Prevention Institute, University of Zurich, Zurich, Switzerland):2199–206.
70.
Dankel SJ, Loenneke JP, Loprinzi PD. Participation in muscle-strengthening activities as an alternative method for the prevention of multimorbidity. Preventive Medicine. 2015;81:54–7.
71.
Dankel SJ, Loenneke JP, Loprinzi PD. Combined Associations of Muscle-Strengthening Activities and Accelerometer-Assessed Physical Activity on Multimorbidity: Findings From NHANES. American Journal of Health Promotion. 2017;31(4):274–7.
72.
Dankel SJ, Loenneke JP, Loprinzi PD. The Impact of Overweight/Obesity Duration and Physical Activity on Medical Multimorbidity: Examining the WATCH Paradigm. American Journal of Health Promotion. 2018;32(8):1747–50.
73.
Dasgupta K, Rosenberg E, Joseph L, Trudeau L, Garfield N, Chan D, et al. Carotid femoral pulse wave velocity in type 2 diabetes and hypertension: capturing arterial health effects of step counts. Journal of Hypertension. 2017;35(5):1061–9.
74.
de Groot IB, Bussmann JB, Stam HJ, Verhaar JA, de Groot IB, Bussmann JB, et al. Actual everyday physical activity in patients with end-stage hip or knee osteoarthritis compared with healthy controls. Osteoarthritis & Cartilage. 2008;16(4):436–42.
75.
De Paula T, Viana L, Zucatti A, Leitao C, Gross J, Azevedo M. Dash diet and walking reduced ambulatory blood pressure values in patients with type 2 diabetes and uncontrolled hypertension. Diabetes. 2014;63:A120-A121.
76.
De Rooij B.H., Van Der Berg J.D., Van Der Kallen C.J.H., Savelberg H.H.C.M., Schaper N.C., Dagnelie P.C., et al. Sedentary time in ‘metabolically healthy’ versus ‘metabolically unhealthy’ obese and non-obese individuals. European Journal of Epidemiology. 2015;30(8):748–9.
77.
De Rooij B.H., Van Der Berg J.D., Van Der Kallen C.J.H., Schram M.T., Savelberg H.H.C.M., Schaper N.C., et al. Physical activity and sedentary behavior in metabolically healthy versus unhealthy obese and non-obese individuals - The Maastricht study. PLoS ONE. 2016;11(5):e0154358.
78.

Delfino R.J., Tjoa T., Gillen D.L., Staimer N., Polidori A., Arhami M., et al. Traffic-related air pollution and blood pressure in elderly subjects with coronary artery disease. Epidemiology. 2010;21(3):396–404.
79.
Demeyer H, Waschki B, Polkey M, Furlanetto K, Donaire-Gonzalez D, Anto J, et al. The survival effect of physical activity in patients with COPD: every step counts. European respiratory journal. 2017;50(Suppl 61):OA512.
80.
Demeyer H., Costilla-Frias M., Louvaris Z., Gimeno-Santos E., Tabberer M., Ivanoff N., et al. Both moderate and severe exacerbations accelerate physical activity decline in COPD patients. American Journal of Respiratory and Critical Care Medicine. 2017;195((Demeyer, Serra, Garcia-Aymerich) ISGlobal, Center for Research in Environmental Epidemiology, Barcelona, Spain).
81.
Demeyer H., Donaire-Gonzalez D., Gimeno-Santos E., Ramon M.A., DE Battle J., Benet M., et al. Physical Activity Is Associated with Attenuated Disease Progression in Chronic Obstructive Pulmonary Disease. Medicine and science in sports and exercise. 2019;51(5):833–40.
82.
Denkinger M.D., Lukas A., Herbolsheimer F., Peter R., Nikolaus T. Physical activity and other health-related factors predict health care utilisation in older adults. The actife ulm study. Zeitschrift fur Gerontologie und Geriatrie. 2012;45(4):290–7.
83.
Di Marco F, Terraneo S, Roggi MA, Repossi AC, Pellegrino GM, Veronelli A, et al. Physical Activity Impairment in Depressed COPD Subjects. Respiratory Care. 2014;59(5):726–34.
84.
Dillon C.B., McMahon E., O’Regan G., Perry I.J. Associations between physical behaviour patterns and levels of depressive symptoms, anxiety and well-being in middle-aged adults: A cross-sectional study using isotemporal substitution models. BMJ Open. 2018;8(1):e018978.
85.
Dodd J.W., Shrikrishnapalasuriyar D., Hopkinson N.S., Jones P. Physical activity and cognitive function in stable non-hypoxaemic patients with chronic obstructive pulmonary disease (COPD). American Journal of Respiratory and Critical Care Medicine. 2011;183(1 MeetingAbstracts).
86.
Dohrn IM, Welmer AK, Hagströmer M. Accelerometry-assessed physical activity and sedentary time and associations with chronic disease and hospital visits - a prospective cohort study with 15 years follow-up. International Journal of Behavioral Nutrition & Physical Activity. 2019;16(1):N.PAG-N.PAG.
87.
Donaire-Gonzalez D., Gimeno-Santos E., Garcia-Aymerich J. Effect of physical inactivity on COPD exacerbations. European Respiratory Journal. 2013;42(SUPPL. 57).
88.
Duenas-Espin I., Demeyer H., Gimeno-Santos E., Polkey M., Rabinovich R., Dobbels F., et al. Longitudinal effects of anxiety and depression on physical activity in COPD patients. European Respiratory Journal. 2014;44(SUPPL. 58).
89.
Duenas-Espin I., Demeyer H., Gimeno-Santos E., Polkey M.I., Hopkinson N.S., Rabinovich R.A., et al. Depression symptoms reduce physical activity in COPD patients: A prospective multicenter study. International Journal of COPD. 2016;11(1):1287–95.
90.

Dunlop D.D., Song J., Semanik P.A., Sharma L., Bathon J.M., Eaton C., et al. Physical activity is associated with reduced incident disability: Evidence from the osteoarthritis initiative. Arthritis and Rheumatism. 2013;65(SUPPL. 10):S103.
91.
Dunlop D.D., Song J., Semanik P.A., Sharma L., Bathon J.M., B Eaton C., et al. Relation of physical activity time to incident disability in community dwelling adults with or at risk of knee arthritis: Prospective cohort study OPEN ACCESS. BMJ (Online). 2014;348((Dunlop, Song, Sharma, Chang) Institute for Public Health and Medicine Center for Healthcare Studies, Feinberg School of Medicine, Northwestern University, 750 Lakeshore Drive, Chicago, IL 60611, United States):g2472.
92.
Durheim M, Smith P, Babyak M, Mabe S, Martinu T, Welty-Wolf K, et al. Six-minute-walk distance and accelerometry predict outcomes in chronic obstructive pulmonary disease independent of Global Initiative for Chronic Obstructive Lung Disease 2011 Group. Annals of the american thoracic society. 2015;12(3):349-356.
93.
Edwardson CL. Activity-related parenting practices and young people's physical activity [Internet]. 2010 [cited 2021 Mar 1]. Available from: http://hdl.handle.net/10068/971779
94.
Eliason G., Zakrisson A.-B., Piehl-Aulin K., Hurtig-Wennlof A. Physical activity patterns in patients in different stages of chronic obstructive pulmonary disease. COPD: Journal of Chronic Obstructive Pulmonary Disease. 2011;8(5):369–74.
95.
Farr J, Going S, Lohman T, Rankin L, Kasle S, Cornett M, et al. Physical activity levels in patients with early knee osteoarthritis measured by accelerometry. Arthritis and rheumatism. 2008;59(9):1229-1236.
96.
Fawole H.O., Felson D.T., Jafarzadeh S.R., Dell'Isola A., Steultjens M.P., Nevitt M.C., et al. The relation of physical activity with fatigue in persons with symptomatic knee osteoarthritis and its potential mediation by physical function or depressive symptoms: the most study. Osteoarthritis and Cartilage. 2020;28(Supplement 1):S412–3.
97.
Fawole H.O., Riskowski J.L., Dell'Isola A., Steultjens M.P., Chastin S.F., Nevitt M.C., et al. Prospective association of physical activity to follow-up fatigue in knee osteoarthritis: the most study. Osteoarthritis and Cartilage. 2019;27(Supplement 1):S255–6.
98.
Freene N., McManus M., Mair T., Tan R., Davey R. Objectively measured changes in physical activity and sedentary behavior in cardiac rehabilitation: A prospective cohort study. Journal of Cardiopulmonary Rehabilitation and Prevention. 2018;38(6):E5–8.
99.
Full K.M., Malhotra A., Gallo L.C., Kerr J., Arredondo E.M., Natarajan L., et al. Accelerometer-Measured Sleep Duration and Clinical Cardiovascular Risk Factor Scores in Older Women. The journals of gerontology Series A, Biological sciences and medical sciences. 2020;75(9):1771–8.
100.
Full KM, Gabriel K, Whitaker KM, Lewis CE, Sternfeld B, Gibbs BB, et al. Prospective associations of accelerometermeasured physical activity and sedentary time with cardiometabolic multimorbidity in the cardia study. Circulation. 2020;141(SUPPL 1).
101.

Fulton R, Witham M, Greig C, Johnston D, Lang C, Van Der Pol M, et al. A randomised controlled trial of exercise training for older heart failure patients. Age and ageing. 2011;40:ii9-.
102.
Garcia J.M., Cox D., Rice D.J. Association of physiological and psychological health outcomes with physical activity and sedentary behavior in adults with type 2 diabetes. BMJ Open Diabetes Research and Care. 2017;5(1):e000306.
103.
Gardner A.W., Montgomery P.S., Wang M., Shen B. ASSOCIATION BETWEEN MEETING DAILY STEP COUNT GOALS WITH AMBULATORY FUNCTION AND QUALITY OF LIFE IN PATIENTS WITH CLAUDICATION. Journal of vascular surgery. 2020;((Gardner, Montgomery) Department of Physical Medicine&Rehabilitation, Penn State College of Medicine, Hershey, PA; Reynolds Oklahoma Center on Aging, University of Oklahoma Health Sciences Center).
104.
Geidl W., Carl J., Cassar S., Lehbert N., Wittmann M., Wagner R., et al. Classifying the physical activity and sedentary behaviour of persons with COPD before pulmonary rehabilitation: A cluster analysis. European Respiratory Journal. 2019;54(Supplement 63).
105.
Geidl W., Carl J., Cassar S., Lehbert N., Mino E., Wittmann M., et al. Physical activity and sedentary behaviour patterns in 326 persons with COPD before starting a pulmonary rehabilitation: A cluster analysis. Journal of Clinical Medicine. 2019;8(9):1346.
106.
Gilbert A., Lee J., Song J., Semanik P., Ehrlich-Jones L.S., Kwoh C.K., et al. The relationship between self-reported restless sleep and objectively measured physical activity. Arthritis and Rheumatology. 2017;69(Supplement 10).
107.
Gilbert A., Song J., Semanik P.A., Chang R.W., Dunlop D.D. Physical inactivity to activity associated with less decline in physical function. Arthritis and Rheumatology. 2014;66(SUPPL. 10):S28.
108.
Gilbert A.L., Lee J., Song J., Semanik P.A., Ehrlich-Jones L.S., Kwoh C.K., et al. Relationship Between Self-Reported Restless Sleep and Objectively Measured Physical Activity in Adults With Knee Osteoarthritis. Arthritis Care and Research. 2018;((Gilbert) University of North Carolina, Chapel Hill, United States).
109.
Gilbert A, Lee J, Ehrlich-Jones L, Semanik P, Song J, Pellegrini C, et al. A randomized trial of a motivational interviewing intervention to increase lifestyle physical activity and improve self-reported function in adults with arthritis. Seminars in arthritis and rheumatism. 2018;47(5):732-740.
110.
Gimeno-Santos E., Arbillaga-Etxarri A., Barberan-Garcia A., Benages-Albert M., Benet M., Dadvand P., et al. Social and environmental determinants of physical activity in patients with chronic obstructive pulmonary disease (COPD). European Respiratory Journal. 2016;48(Supplement 60).
111.
Gomes R da S, Barbosa AR, Meneghini V, Confortin SC, d'Orsi E, Rech CR, et al. Association between chronic diseases, multimorbidity and insufficient physical activity among older adults in southern Brazil: a cross-sectional study. Sao Paulo Medical Journal. 2020;138(6):545–53.

112.
Gomes R da S, Barbosa AR, Meneghini V, Confortin SC, d'Orsi E, Rech CR, et al. Association between chronic diseases, multimorbidity and insufficient physical activity among older adults in southern Brazil: a cross-sectional study. Sao Paulo Medical Journal. 2020 Dec;138(6):545–53.
113.
Gordon-Larsen P., Boone-Heinonen J., Sidney S., Sternfeld B., Jacobs Jr. D.R., Lewis C.E. Active commuting and cardiovascular disease risk: The CARDIA study. Archives of Internal Medicine. 2009;169(13):1216–23.
114.
Gulart AA, Munari AB, Santos Silva IJC, Alexandre HF, Karloh M, Mayer AF. Baseline characteristics associated to improvement of patients with COPD in physical activity in daily life level after pulmonary rehabilitation. Respiratory medicine. 2019;151(8908438, rme):142–7.
115.
Hagstromer M., Kwak L., Oja P., Sjostrom M. A 6 year longitudinal study of accelerometer-measured physical activity and sedentary time in Swedish adults. Journal of Science and Medicine in Sport. 2015;18(5):553–7.
116.
Hains-Monfette G, Atoui S, Needham Dancause K, Bernard P. Device-Assessed Physical Activity and Sedentary Behaviors in Canadians with Chronic Disease(s): Findings from the Canadian Health Measures Survey. Sports (Basel, Switzerland) [Internet]. 2019;7(5). Available from: https://res.mdpi.com/d_attachment/sports/sports-07-00113/article_deploy/sports-07-00113.pdf
117.
Hains-Monfette G, Atoui S, Needham Dancause K, Bernard P. Device-Assessed Physical Activity and Sedentary Behaviors in Canadians with Chronic Disease(s): Findings from the Canadian Health Measures Survey. Sports (Basel). 2019 May 16;7(5).
118.
Hamasaki H., Noda M., Moriyama S., Yoshikawa R., Katsuyama H., Sako A., et al. Daily physical activity assessed by a triaxial accelerometer is beneficially associated with waist circumference, serum triglycerides, and insulin resistance in Japanese patients with prediabetes or untreated early type 2 diabetes. Journal of Diabetes Research. 2015;2015((Hamasaki, Moriyama, Yoshikawa, Katsuyama, Sako, Mishima, Yanai) Department of Internal Medicine, National Center for Global Health, Medicine Kohnodai Hospital, Chiba 272-8516, Japan):526201.
119.
Hartman JE, Boezen HM, de Greef MH, ten Hacken NH. Physical and Psychosocial Factors Associated With Physical Activity in Patients With Chronic Obstructive Pulmonary Disease. Archives of Physical Medicine & Rehabilitation. 2013;94(12):2396-2402.e7.
120.
Hataji O., Nishii Y., Sakaguchi T., Saiki H., Ito K., D'Alessandro-Gabazza C., et al. Smart watch for monitoring physical activity in patients with chronic obstructive pulmonary disease. European Respiratory Journal. 2016;48(Supplement 60).
121.
Healy GN, Wijndaele K, Dunstan DW, Shaw JE, Salmon J, Zimmet PZ, et al. Objectively measured sedentary time, physical activity, and metabolic risk: the Australian Diabetes, Obesity and Lifestyle Study (AusDiab). Diabetes care. 2008;31(2):369–71.
122.
Healy G, Winkler E, Brakenridge C, Reeves M, Eakin E. Accelerometer-derived sedentary and physical activity time in overweight/obese adults with type 2 diabetes: cross-sectional

associations with cardiometabolic biomarkers. PloS one [Internet]. 2015;10(3). Available from: https://storage.googleapis.com/plos-corpus-prod/10.1371/journal.pone.0119140/1/pone.0119140.pdf?X-Goog-Algorithm=GOOG4-RSA-SHA256&X-Goog-Credential=wombat-sa%40plos-prod.iam.gserviceaccount.com%2F20210625%2Fauto%2Fstorage%2Fgoog4_request&X-Goog-Date=20210
123.
Helgadottir B., Forsell Y., Ekblom O. Physical activity patterns of people affected by depressive and anxiety disorders as measured by accelerometers: A cross-sectional study. PLoS ONE. 2015;10(1):e0115894.
124.
Hermida R.C., Ayala D.E., Mojon A., Fernandez J.R. Ambulatory blood pressure thresholds for diagnosing hypertension in type 2 diabetes based on cardiovascular outcomes. Journal of Clinical Hypertension. 2012;14(SUPPL. 1).
125.
Hershman S.G., Bot B.M., Shcherbina A., Doerr M., Moayedi Y., Pavlovic A., et al. Physical activity, sleep and cardiovascular health data for 50,000 individuals from the MyHeart Counts Study. Scientific data. 2019;6(1):24.
126.
Hinrichs T, Bücker B, Klaaßen-Mielke R, Brach M, Wilm S, Platen P, et al. Home-Based Exercise Supported by General Practitioner Practices: Ineffective in a Sample of Chronically Ill, Mobility-Limited Older Adults (the HOMEfit Randomized Controlled Trial). J Am Geriatr Soc. 2016 Nov;64(11):2270–9.
127.
Hirano T., Mori H., Hamada K., Suetake R., Murata Y., Oishi K., et al. Association between patient reported outcome measures and physical activity in obstructive respiratory disease. Respirology. 2019;24(Supplement 2):162–3.
128.
Holber J.P., Abebe K., Anderson A.M., Jakicic J.M., Ramani R., Rollman B.L., et al. Mood symptomsare not associated with objectively assessed physical activityamong patients recently hospitalized with systolic heart failure. Journal of General Internal Medicine. 2018;33(2 Supplement 1):264–5.
129.
Holber J.P., Huang Y., Abebe K.Z., Anderson A., Jakicic J.M., Rollman B.L., et al. Does neighborhood walkability and area deprivation correlate with physical activity among recently hospitalized patients with systolic heart failure and co-morbid depression? Psychosomatic Medicine. 2019;81(4):A140–1.
130.
Honda T., Chen S., Kishimoto H., Narazaki K., Kumagai S. Identifying associations between sedentary time and cardio-metabolic risk factors in working adults using objective and subjective measures: a cross-sectional analysis. BMC public health. 2014;14((Kumagai) Department of Behavior and Health Sciences, Graduate School of Human-Environment Studies, Kyushu University, 6-1 Kasuga kouen, Kasuga City, Fukuoka Prefecture 816-8580, Japan. shuzo@ihs.kyushu-u.ac.jp):1307.
131.
Hospes G, Bossenbroek L, Ten Hacken N, van Hengel P, de Greef M. Enhancement of daily physical activity increases physical fitness of outclinic COPD patients: results of an exercise counseling program. Patient education and counseling. 2009;75(2):274-278.

132.
Houle J, Vadeboncoeur N, Doyon O, Campagna L, Diaz A, Poirie P. Influence of a counseling and pedometer-based activity program on physical activity behavior and risk factors after a coronary heart disease event (paradis project). Journal of cardiopulmonary rehabilitation and prevention. 2008;28(5):336-.
133.
Hoaas H., Zanaboni P., Hjalmarsen A., Morseth B., Dinesen B., Burge A.T., et al. Seasonal variations in objectively assessed physical activity among people with COPD in two Nordic countries and Australia: A cross-sectional study. International Journal of COPD. 2019;14((Hoaas, Zanaboni) Norwegian Centre for E-health Research, University Hospital of North Norway, Tromso, Norway):1219–28.
134.
Hsueh M.-C., Stubbs B., Lai Y.-J., Sun C.-K., Chen L.-J., Ku P.-W. A dose response relationship between accelerometer assessed daily steps and depressive symptoms in older adults: a two-year cohort study. Age and ageing. 2020;((Hsueh) Graduate Institute of Sport Pedagogy, University of Taipei, Taipei 111, Taiwan (Republic of China)).
135.
Huang B.-H., Inan-Eroglu E., Hamer M., Stamatakis E. Joint associations of device-measured physical activity and sleep duration with cardiometabolic health in the 1970 British Cohort Study. Journal of Science and Medicine in Sport. 2020;23(12):1191–6.
136.
Hubert D., Rouquette A., Cormier C., Neveux N., Fawzia A., Chapron J., et al. Determinants of physical activity in adults with cystic fibrosis. Pediatric Pulmonology. 2016;51(Supplement 45):375–6.
137.
Huffman K, Sun JL, Thomas L, Bales C, Califf R, Yates T, et al. Impact of baseline physical activity and diet behavior on metabolic syndrome in a pharmaceutical trial: results from NAVIGATOR. Metabolism: clinical and experimental. 2014;63(4):554-561.
138.
Huijnen IP, Verbunt JA, Peters ML, Delespaul P, Kindermans HP, Roelofs J, et al. Do depression and pain intensity interfere with physical activity in daily life in patients with Chronic Low Back Pain? Pain (03043959). 2010;150(1):161–6.
139.
Huo X, Krumholz HM, Bai X, Spatz ES, Ding Q, Horak P, et al. Effects of Mobile Text Messaging on Glycemic Control in Patients With Coronary Heart Disease and Diabetes Mellitus: A Randomized Clinical Trial. Circ Cardiovasc Qual Outcomes. 2019 Sep;12(9):e005805.
140.
Hur S., Guler S.A., Camp P.G., Guenette J.A., Khalil N., Ryerson C.J. Prevalence and impact of extrapulmonary deficits on daily physical activity in fibrotic interstitial lung disease. American Journal of Respiratory and Critical Care Medicine. 2018;197(MeetingAbstracts).
141.
Huxley C.J., Sturt J., Dale J., Griffiths F. A mixed-method exploration of the relationships between physical activity, self-efficacy and distress in people with Type 2 diabetes. Diabetic Medicine. 2013;30(SUPPL. 1):129.
142.
Ichinose M., Minakata Y., Motegi T., Takahashi T., Seki M., Sugaya S., et al. A non-interventional, cross-sectional study to evaluate factors relating to daily step counts and physical activity in Japanese patients with chronic obstructive pulmonary disease: Step COPD.

International Journal of COPD. 2020;15((Ichinose) Academic Center of Osaki Citizen Hospital, Osaki 989-6183, Japan):3385–96.
143.
Idowu O, Adeniyi A. Efficacy of Graded Activity with and without Daily-Monitored-Walking on Pain and Back Endurance among Patients with Concomitant Low-Back Pain and Type-2 Diabetes: a Randomized Trial. Ethiopian journal of health sciences. 2020;30(2):233-242.
144.
Iwakura M., Okura K., Kawagoshi A., Shiata K., Satake M., Sugawara K., et al. Relationships between balance and physical activity measured by an accelerometer in elderly COPD patients. European Respiratory Journal. 2016;48(Supplement 60).
145.
Jakicic J.M., Fulton J.E., Lang W., Walkup M.P. Association of the change in physical activity and cardiovascular disease outcomes in the look ahead trial. Circulation. 2018;137(Supplement 1).
146.
Jardim J.R., Piazza M., Carvalho A.K., Ivanaga I.T., Nascimento O.A. Assessment of physical activity by pedometer in patients with chronic obstructive pulmonary disease (COPD) In an emerging country. American Journal of Respiratory and Critical Care Medicine. 2011;183(1 MeetingAbstracts).
147.
Jefferis B.J., Sartini C., Shiroma E., Whincup P.H., Wannamethee S.G., Lee I.-M. Duration and breaks in sedentary behaviour: accelerometer data from 1566 community-dwelling older men (British Regional Heart Study). British journal of sports medicine. 2015;49(24):1591–4.
148.
Jefferis BJ, Sartini C, Lee IM, Choi M, Amuzu A, Gutierrez C, et al. Adherence to physical activity guidelines in older adults, using objectively measured physical activity in a population-based study. BMC Public Health. 2014;14(1):382–382.
149.
Jehn M., Schmidt-Trucksass A., Meyer A., Schindler C., Tamm M., Stolz D. Association of daily physical activity volume and intensity with COPD severity. Respiratory Medicine. 2011;105(12):1846–52.
150.
Jeong J.-N., Kim S.-H., Park K.-N. Relationship between objectively measured lifestyle factors and health factors in patients with knee osteoarthritis: The STROBE Study. Medicine. 2019;98(26):e16060.
151.
Joven M., Croghan I., Schroeder D., Quigg S., Ebbert J., Takahashi P. Predictors of sedentary status in overweight and obese patients with multiple chronic conditions, a cohort study. European Geriatric Medicine. 2016;7(Supplement 1):S87.
152.
Joven MH, Croghan IT, Quigg SM, Ebbert JO, Takahashi PY. Predictors of sedentary status in overweight and obese patients with multiple chronic conditions: a cohort study. Pragmatic and observational research. 2017;8(101688693):203–9.
153.
JPRN-UMIN000021613. Effects of a combined pedometer and home blood pressure monitoring program on blood pressure control in community-dwelling elderly adults with hypertension and diabetes. http://www.who.int/trialsearch/Trial2.aspx?TrialID=JPRN-UMIN000021613. 2016;
154.

Kakar R.S., Lomond K. Can COVID-19 Lead to Another Pandemic of Back Pain? Archives of Physical Medicine and Rehabilitation. 2020;101(12):e132.
155.
Kantorowski A, Wan E, Homsy D, Kadri R, Richardson C, Moy M. Determinants and outcomes of change in physical activity in COPD. ERJ open research. 2018;4(3) (no pagination).
156.
Kao M.C.J., Jarosz R., Goldin M., Patel A., Smuck M. Determinants of physical activity in America: A first characterization of physical activity profile using the National Health and Nutrition Examination Survey (NHANES). PM and R. 2014;6(10):882–92.
157.
Khambaty T, Moncrieft A, Llabre M, McCalla J, Schneiderman N. Lifestyle intervention components as predictors of weight loss in low income, minority patients with type 2 diabetes: results of the calm-d randomized controlled trial. Psychosomatic medicine. 2016;78(3):A134-.
158.
Kirk A.F., Barnettw J., Leesez G., Mutriey N. Twelve month changes in physical a outcomes following a written or person delivered physical activity consultation in Type 2 diabetes (TIME2ACT): A randomised trial. Diabetic Medicine. 2009;26(SUPPL. 1):167.
159.
Kirk AF. Promoting and maintaining physical activity in people with type 2 diabetes [Internet]. 2003 [cited 2021 Mar 1]. Available from: http://hdl.handle.net/10068/898210
160.
Kloek C, Van Dongen J, Bossen D, Dekker J, Veenhof C. Cost-effectiveness of a blended physiotherapy intervention in patients with hip and/or knee osteoarthritis: a cluster randomised controlled trial. Annals of the rheumatic diseases. 2018;77:1132-.
161.
Klompstra L, Back M, Stromberg A, Piepoli M, Jaarsma T. Objectively Measured Physical Activity in Patients with Heart Failure. Circulation [Internet]. 2020;142(SUPPL 3). Available from: https://www.cochranelibrary.com/central/doi/10.1002/central/CN-02259486/full
162.
Kolbasi E., Tamer D., Gurbuz M., Caglar A., Aytar A. Effects of pedometer use on physical fitness in subjects with knee osteoarthritis. Fizyoterapi Rehabilitasyon. 2015;26(3):S53.
163.
Koolhaas C.M., Van Rooij F.J.A., Schoufour J.D., Cepeda M., Tiemeier H., Brage S., et al. Objective measures of activity in the elderly: Distribution and associations with demographic and health factors. Maturitas. 2017;103((Koolhaas, Van Rooij, Schoufour, Cepeda, Tiemeier, Brage, Franco) Erasmus MC, Netherlands):91–2.
164.
Kostorz S., Jastrzebski D., Sikora M., Zebrowska A., Margas A., Stepanik D., et al. Predominance of comorbidities in the detriment of daily activity in sarcoidosis patients. Advances in Experimental Medicine and Biology. 2018;1040((Kostorz, Jastrzebski, Margas, Stepanik, Swinder, Ziora) School of Medicine with the Division of Dentistry, Department of Lung Diseases and Tuberculosis, Medical University of Silesia, 1 Koziolka Street, Zabrze 41-803, Poland):7–12.
165.
Koukouvou G, Kouidi E, Iacovides A, Konstantinidou E, Kaprinis G, Deligiannis A. Quality of life, psychological and physiological changes following exercise training in patients with chronic heart failure. J Rehabil Med. 2004 Jan;36(1):36–41.
166.

Kraus W, Califf R, Tuomilehto J, Sun JL, Thomas L, Yates T, et al. Relations between baseline physical activity by pedometer counts and future cardiovascular events in the navigator study. Circulation. 2012;126(21 SUPPL. 1).
167.
Kujala U.M., Hautasaari P., Vaha-Ypya H., Waller K., Lindgren N., Iso-Markku P., et al. Chronic diseases and objectively monitored physical activity profile among aged individuals-a cross-sectional twin cohort study. Annals of Medicine. 2019;51(1):78–87.
168.
Kulcu DG, Kurtais Y, Tur BS, Gülec S, Seckin B. The effect of cardiac rehabilitation on quality of life, anxiety and depression in patients with congestive heart failure. A randomized controlled trial, short-term results. Eura Medicophys. 2007 Dec;43(4):489–97.
169.
LaCroix A, Bellettiere J, Rillamas-Sun E, Di C, Evenson K, Lewis C, et al. Association of Light Physical Activity Measured by Accelerometry and Incidence of Coronary Heart Disease and Cardiovascular Disease in Older Women. JAMA network open. 2019;2(3):e190419-.
170.
Lamers F., Difrancesco S., Riese H., Van Hemert B., Schoevers R., Penninx B. Objectively measured physical activity and sleep and its associations with depressive and anxiety disorders. Biological Psychiatry. 2018;83(9 Supplement 1):S174.
171.
Landi F., Calvani R., Picca A., Tosato M., Martone A.M., Ortolani E., et al. Body mass index is strongly associated with hypertension: Results from the longevity check-up 7+ study. Nutrients. 2018;10(12):1976.
172.
Langer D., Pitta F., Troosters T., Burtin C., Decramer M., Gosselink R. Quantifying physical activity in COPD: Different measures for different purposes. Thorax. 2009;64(5):458.
173.
Larson J.L., Covey M.K., Kapella M.C. Effects of self-efficacy enhancing intervention to increase physical activity in people with copd. American Journal of Respiratory and Critical Care Medicine. 2011;183(1 MeetingAbstracts).
174.
Lee J, Song J, Hootman JM, Semanik PA, Chang RW, Sharma L, et al. Obesity and other modifiable factors for physical inactivity measured by accelerometer in adults with knee osteoarthritis. Arthritis Care & Research. 2013;65(1):53–61.
175.
Lee J., Chang R.W., Manheim L., Semanik P.A., Song J., Dunlop D.D. Sedentary behavior and functional performance among participants in the osteoarthritis initiative (OAI). Arthritis and Rheumatism. 2011;63(10 SUPPL. 1).
176.
Lee J., Song J., Chang R.W., Ehrlich- Jones L.S., Semanik P.A., Sohn M.-W., et al. Less time spent in sedentary behavior is associated with better future physical function: Objective data from the osteoarthritis initiative. Arthritis and Rheumatism. 2013;65(SUPPL. 10):S459–60.
177.
Li Y.-N., Shapiro B., Kim J.C., Zhang M., Porszasz J., Bross R., et al. Association between quality of life and anxiety, depression, physical activity and physical performance in maintenance hemodialysis patients. Chronic Diseases and Translational Medicine. 2016;2(2):110–9.
178.

Limbach M., Wittmann M., Lehbert N., Geidl W., Carl J., Pfeifer K., et al. Pulmonary rehabilitation in COPD: influence of comorbidities on physical activity. European Respiratory Journal. 2020;56(Supplement 64).
179.
Linde C, Tang A, Cowie M, Bergemann T, Abraham W. Physical activity measured with implanted devices predicts heart failure outcomes. European journal of heart failure. 2017;19:347-.
180.
Loprinzi P.D. Health-Enhancing Multibehavior and Medical Multimorbidity. Mayo Clinic Proceedings. 2015;90(5):624–32.
181.
Loprinzi P.D. Sedentary behavior and medical multimorbidity. Physiology and Behavior. 2015;151((Loprinzi) Center for Health Behavior Research, Department of Health, Exercise Science and Recreation Management, The University of Mississippi, MS, United States):395–7.
182.
Loprinzi P.D. Accelerometer-determined physical activity and mortality in a national prospective cohort study of adults at high risk of a first atherosclerotic cardiovascular disease event. International Journal of Cardiology. 2016;202((Loprinzi) Center for Behavioral Research, Department of Health, Exercise Science, and Recreation Management, University of Mississippi, 229 Turner Center, University, MS 38677, United States):417–8.
183.
Loprinzi P.D. Health behavior characteristics and all-cause mortality. Preventive Medicine Reports. 2016;3((Loprinzi) Jackson Heart Study Vanguard Center of Oxford, Department of Health, Exercise Science and Recreation Management, Physical Activity Epidemiology Laboratory, The University of Mississippi, University MS, 229 Turner Center, Oxford, MS 38677, Unite):276–8.
184.
Loprinzi P.D. Physical activity and peripheral arterial disease among patients with coronary artery disease or congestive heart failure. International Journal of Cardiology. 2016;207((Loprinzi) University of Mississippi, Jackson Heart Study Vanguard Center of Oxford, Center for Health Behavior Research, School of Applied Sciences, Department of Health, Exercise Science, and Recreation Management, 229 Turner Center, University, MS 3867):110–1.
185.
Loprinzi P.D. Associations between bouted and non-bouted physical activity on multimorbidity. Clinical Physiology and Functional Imaging. 2017;37(6):782–4.
186.
Loprinzi P.D., Abbott K. Association of diabetic peripheral arterial disease and objectively-measured physical activity: NHANES 2003-2004. Journal of Diabetes and Metabolic Disorders. 2014;13(1):63.
187.
Loprinzi P.D., Addoh O. Association of Objectively Measured Physical Activity With Objectively Measured Visual Acuity Among a Population-Based Sample of Patients With Coronary Artery Disease and Congestive Heart Failure: the Cardio-ocular Paradigm. Mayo Clinic Proceedings. 2016;91(6):820.
188.
Loprinzi P.D., Franz C., Hager K.K. Accelerometer-assessed physical activity and depression among U.S. adults with diabetes. Mental Health and Physical Activity. 2013;6(2):79–82.
189.

Loprinzi P.D., Sheffield J., Tyo B.M., Fittipaldi-Wert J. Accelerometer-determined physical activity, mobility disability, and health. Disability and health journal. 2014;7(4):419–25.
190.
Loprinzi PD. Accelerometer-Determined Sedentary and Physical Activity Estimates Among Older Adults With Diabetes: Considerations by Demographic and Comorbidity Characteristics. Journal of Aging & Physical Activity. 2008;22(3):432–40.
191.
Loprinzi PD. Accelerometer-Determined Sedentary and Physical Activity Estimates Among Older Adults With Diabetes: Considerations by Demographic and Comorbidity Characteristics. Journal of Aging & Physical Activity. 2014;22(3):432–40.
192.
Loprinzi PD. Light-Intensity Physical Activity and Medical Multimorbidity. Southern Medical Journal. 2016;109(3):174–7.
193.
Loprinzi PD. Light-Intensity Physical Activity and Medical Multimorbidity. South Med J. 2016 Mar;109(3):174–7.
194.
Loprinzi PD, Addoh O. Association of Objectively Measured Physical Activity With Objectively Measured Visual Acuity Among a Population-Based Sample of Patients With Coronary Artery Disease and Congestive Heart Failure: the Cardio-ocular Paradigm. Mayo Clinic Proceedings. 2016 Jun 1;91(6):820.
195.
Lores V., Garcia-Rio F., Rojo B., Alcolea S., Mediano O. Recording the daily physical activity of COPD patients with an accelerometer: An analysis of agreement and repeatability. Archivos de Bronconeumologia. 2006;42(12):627–32.
196.
Lotzke H., Jakobsson M., Gutke A., Hagstromer M., Brisby H., Hagg O., et al. Patients with severe low back pain exhibit a low level of physical activity before lumbar fusion surgery: A cross-sectional study 11 Medical and Health Sciences 1117 Public Health and Health Services 11 Medical and Health Sciences 1103 Clinical Sciences. BMC Musculoskeletal Disorders. 2018;19(1):365.
197.
Ly K., Hajna S., Da Costa D., Dasgupta K. The impact of walking on depression in adults with type 2 diabetes. Diabetes. 2011;60(SUPPL. 1):A219–20.
198.
Manas A., Alcazar J., Rodriguez-Lopez C., Rodriguez-Gomez I., Guadalupe-Grau A., Alfaro-Acha A., et al. Functional ability: An effective biomarker of healthy ageing independently of multimorbidity. Maturitas. 2017;100((Manas, Alcazar, Rodriguez-Lopez, Rodriguez-Gomez, Alegre, Ara) GENUD Toledo Research Group (Toledo, Spain), Toledo, Spain):198–9.
199.
Manheim L, Song J, Chang R, Dunlop D. Relationship between physical activity and health-related utility in Knee Osteoarthritis patients. Arthritis and rheumatism. 2010;62:1558-.
200.
Manjoo P., Joseph L., Pilote L., Dasgupta K. Waist to hip mediates inverse association of walking with A1C but not of walking with blood pressure among women with type 2 diabetes. Canadian Journal of Diabetes. 2009;33(3):274.
201.

Mantoani L, Mckinstry B, Mcnarry S, Mullen S, Begg S, Saini P, et al. Physical activity enhancing programme (PAEP) in COPD – a randomised controlled trial. European respiratory journal. 2018;52(suppl 62):OA1986.
202.
Mantovani A.M., Duncan S., Codogno J.S., Lima M.C., Fernandes R.A. Different Amounts of Physical Activity Measured by Pedometer and the Associations With Health Outcomes in Adults. Journal of physical activity & health. 2016;13(11):1183–91.
203.
Marigliano E., Fahs P.S., Ludden C. Walking for Heart Health: A Study of Adult Women in Rural New York. Creative nursing. 2016;22(4):268–75.
204.
Marsaux CFM, Celis-Morales C, Hoonhout J, Claassen A, Goris A, Forster H, et al. Objectively Measured Physical Activity in European Adults: Cross-Sectional Findings from the Food4Me Study. PLOS ONE. 2016 Mar 21;11(3):e0150902.
205.
Martinez C.H., Moy M.L., Nguyen H.Q., Cohen M., Kadri R., Roman P., et al. Beliefs about disease controllability are related with baseline physical activity and with longitudinal change on physical activity among COPD patients. American Journal of Respiratory and Critical Care Medicine. 2014;189(MeetingAbstracts).
206.
Matkovic Z., Cvetko D., Rahelic D., Lopez C.E., Tudoric N., Miravitlles M. Evaluation of nutritional status, disease severity and anxiety/depression in COPD patients with different exercise capacity. European Respiratory Journal. 2016;48(Supplement 60).
207.
Matkovic Z., Cvetko D., Rahelic D., Esquinas C., Zarak M., Miravitlles M., et al. Nutritional status and physical activity in patients with COPD, and factors associated with malnutrition. European Respiratory Journal. 2019;54(Supplement 63).
208.
Matura L., Fritz J.S., Smith K.A., Vaidya A., Patel M., Pinder D., et al. Symptoms and physical activity in pulmonary arterial hypertension. American Journal of Respiratory and Critical Care Medicine. 2015;191(MeetingAbstracts).
209.
Mavroeidi A. Influence of habitual physical activity on bone mass of postmenopausal women [Internet]. 2005 [cited 2021 Mar 1]. Available from: http://hdl.handle.net/10068/925641
210.
McCullagh R., Darren D., Horgan N.F., Timmons S. Factors Associated With Walking in Older Medical Inpatients. Archives of Rehabilitation Research and Clinical Translation. 2020;2(1):100038.
211.
McGlone S., Venn A., Walters E.H., Wood-Baker R. Physical activity, spirometry and quality-of-life in chronic obstructive pulmonary disease. COPD: Journal of Chronic Obstructive Pulmonary Disease. 2006;3(2):83–8.
212.
McKeough Z., Leung R., McDonald C., Jenkins S., Holland A., Hill K., et al. Exercise capacity and activity levels in people with COPD who experience exerciseinduced oxygen desaturation. European Respiratory Journal. 2016;48(Supplement 60).
213.

Mesquita R., Meijer K., Pitta F., Azcuna H., Goertz Y.M.J., Essers J.M.N., et al. Changes in physical activity and sedentary behaviour following pulmonary rehabilitation in patients with COPD. Respiratory Medicine. 2017;126((Mesquita, Goertz, Wouters, Spruit) Department of Research&Education, CIRO, Center of Expertise for Chronic Organ Failure, Horn, Netherlands):122–9.
214.
Mesquita R., Nakken N., Janssen D.J.A., Van Den Bogaart E.H.A., Delbressine J.M.L., Essers H., et al. Physical activity and sedentary behaviour in patients with COPD and their resident loved ones. European Respiratory Journal. 2016;48(Supplement 60).
215.
Mesquita R., Spina G., Pitta F., Donaire-Gonzalez D., Deering B.M., Patel M.S., et al. Physical activity patterns and clusters in 1001 patients with COPD. Chronic Respiratory Disease. 2017;14(3):256–69.
216.
Michishita R., Shono N., Kasahara T., Katoku M., Tsuruta T. The possible influence of osteoarthritis of the knee on the accumulation of coronary risk factors in postmenopausal obese women. Obesity Research and Clinical Practice. 2008;2(1):29–34.
217.
Miller C. A symptom management intervention in diabetic coronary artery bypass graft patients. 2005;(University of Nebras):-236.
218.
Miller G.D., Jakicic J.M., Rejeski W.J., Whit-Glover M.C., Lang W., Walkup M.P., et al. Effect of varying accelerometry criteria on physical activity: The look AHEAD study. Obesity. 2013;21(1):32–44.
219.
Minakata Y., Morishita Y., Ichikawa T., Akamatsu K., Hirano T., Nakanishi M., et al. Effects of pharmacologic treatment based on airflow limitation and breathlessness on daily physical activity in patients with chronic obstructive pulmonary disease. International Journal of COPD. 2015;10((Minakata) Department of Respiratory Medicine, National Hospital Organization Wakayama Hospital, Mihama-cho, Hidaka-gun, Wakayama, Japan):1275–82.
220.
Miura M., Yatsu T., Takita K., Abe M., Ito A., Otsuka T., et al. Simple daily step count predicts long-term changes in arterial stiffness in symptomatic COPD patients. European Respiratory Journal. 2016;48(Supplement 60).
221.
Moore R, Berlowitz D, Denehy L, Jackson B, McDonald C. Comparison of pedometer and activity diary for measurement of physical activity in chronic obstructive pulmonary disease. Journal of cardiopulmonary rehabilitation and prevention. 2009;29(1):57-61.
222.
Morcos M.W., Teeter M.G., Somerville L.E., Lanting B. Correlation between hip osteoarthritis and the level of physical activity as measured by wearable technology and patient-reported questionnaires. Journal of Orthopaedics. 2020;20((Morcos, Teeter, Lanting) Bone and Joint Institute, Western University, London, Ontario, Canada):236–9.
223.
Moy M.L., Weston N.A., Wilson E., Richardson C.R. An internet-mediated walking program and pedometer increase walking in COPD. American Journal of Respiratory and Critical Care Medicine. 2012;185(MeetingAbstracts).
224.

Murata S, Doi T, Sawa R, Nakamura R, Isa T, Ebina A, et al. Association Between Objectively Measured Physical Activity and the Number of Chronic Musculoskeletal Pain Sites in Community-Dwelling Older Adults. Pain Medicine. 2019 Apr 1;20(4):717–23.
225.
Murphy S, Schepens Niemiec S, Lyden A, Kratz A. Pain, Fatigue, and Physical Activity in Osteoarthritis: the Moderating Effects of Pain- and Fatigue-Related Activity Interference. Archives of physical medicine and rehabilitation. 2016;97(9):S201-S209.
226.
Murphy SL, Alexander NB, Levoska M, Smith DM. Relationship between fatigue and subsequent physical activity among older adults with symptomatic osteoarthritis. Arthritis Care & Research. 2013;65(10):1617–24.
227.
Murphy SL, Kratz AL, Williams DA, Geisser ME. The Association between Symptoms, Pain Coping Strategies, and Physical Activity Among People with Symptomatic Knee and Hip Osteoarthritis. Frontiers in psychology. 2012;3(101550902):326.
228.
Nawrocka A, Niestrój-Jaworska M, Mynarski A, Polechoński J. Association Between Objectively Measured Physical Activity And Musculoskeletal Disorders, And Perceived Work Ability Among Adult, Middle-Aged And Older Women [Internet]. Vol. 14, Clinical Interventions in Aging. Dove Press; 2019 [cited 2021 Mar 1]. p. 1975–83. Available from: https://www.dovepress.com/association-between-objectively-measured-physical-activity-and-musculo-peer-reviewed-article-CIA
229.
NCT02910544. Weight Loss and Physical Activity in Overweight/Obese Individuals With Knee Osteoarthritis. https://clinicaltrials.gov/show/NCT02910544. 2016;
230.
Neumannova K., Koblizek V., Kovacikova Z., Novotna B., Plutinsky M., Musilova P., et al. Daily physical activity in severe chronic obstructive pulmonary disease (COPD) according to new gold classification and COPD phenotypes-first results from the Czech multicentre research database of COPD. American Journal of Respiratory and Critical Care Medicine. 2015;191(MeetingAbstracts).
231.
Ng C, Jenkins S, Cecins N, Eastwood P, Hill K. The effect of using a wheeled walker on physical activity in people with COPD: preliminary data. Respirology. 2012;17(Suppl 1):60.
232.
Ng L, Jenkins S, Cecins N, Eastwood P, Hill K. A wheeled walker improves physical activity in chronic obstructive pulmonary disease. Physiotherapy (united kingdom). 2015;101:eS1084.
233.
Nguyen HQ, Fan VS, Herting J, Lee J, Fu M, Chen Z, et al. Patients with COPD with higher levels of anxiety are more physically active. CHEST. 2013;144(1):145–51.
234.
Nolan C, Maddocks M, Canavan J, Jones S, Delogu V, Kaliaraju D, et al. Pedometer Step Count Targets during Pulmonary Rehabilitation in Chronic Obstructive Pulmonary Disease. A Randomized Controlled Trial. American journal of respiratory and critical care medicine. 2017;195(10):1344-1352.
235.

O'Donnell J, Smith-Byrne K, Velardo C, Conrad N, Salimi-Khorshidi G, Doherty A, et al. Self-reported and objectively measured physical activity in people with and without chronic heart failure: UK Biobank analysis. Open Heart. 2020;e001099–e001099.
236.
O'Donnell J, Smith-Byrne K, Velardo C, Conrad N, Salimi-Khorshidi G, Doherty A, et al. Self-reported and objectively measured physical activity in people with and without chronic heart failure: UK Biobank analysis. Open Heart. 2020;e001099–e001099.
237.
Okely J.A., Cukic I., Shaw R.J., Chastin S.F., Dall P.M., Deary I.J., et al. Positive and negative well-being and objectively measured sedentary behaviour in older adults: evidence from three cohorts. BMC geriatrics. 2019;19(1):28.
238.
Omar M, Frederiksen P, Videbaek L, Kjaer Poulsen M, Eifer Moeller J, Jensen J, et al. Resting and exercise hemodynamic determinants of daily activity measured by accelerometer in stable heart failure patients. Eur Heart J. 2021;42(SUPPL 1):791.
239.
Oppermann K., Colpani V., Spritzer P.M. Risk factors associated with coronary artery calcification in midlife women: a population-based study. Gynecological Endocrinology. 2019;35(10):904–8.
240.
Ortlieb S., Dias A., Gorzelniak L., Nowak D., Karrasch S., Peters A., et al. Exploring patterns of accelerometry-assessed physical activity in elderly people. International Journal of Behavioral Nutrition and Physical Activity. 2014;11(1):28.
241.
Ortlieb S., Gorzelniak L., Nowak D., Strobl R., Grill E., Thorand B., et al. Associations between multiple accelerometry-assessed physical activity parameters and selected health outcomes in elderly people - Results from the KORA-age study. PLoS ONE. 2014;9(11):e0111206.
242.
Ostlind E, Sant'Anna A, Eek F, Stigmar K, Hansson E. Physical activity pattern and Fitbit use in working patients with hip and/or knee osteoarthritis throughout an intervention: an exploratory descriptive study. Osteoarthritis and cartilage. 2020;28:S167-.
243.
Park J.H., Eun Park J., Sheen S.S., Chung W.Y., Hwang S.C. Cardiovascular co-morbidities of COPD and prism in non-smoking population. Respirology. 2019;24(Supplement 2):39.
244.
Park S., Larson J.L. Physical activity in people with chronic obstructive pulmonary disease, using the national health and nutrition evaluation survey dataset (2003-2006). American Journal of Respiratory and Critical Care Medicine. 2013;187(MeetingAbstracts).
245.
Park SK, Richardson CR, Holleman RG, Larson JL. Physical activity in people with COPD, using the National Health and Nutrition Evaluation Survey dataset (2003–2006). Heart & Lung. 2013;42(4):235–40.
246.
Parker D.C., Hall K., Morey M., Sloane R. Sedentary behavior is associated with elevations in pro-inflammatory biomarkers. Journal of the American Geriatrics Society. 2017;65(Supplement 1):S119.
247.

Peacock O, Western M, Batterham A, Chowdhury E, Stathi A, Standage M, et al. Effect of novel technology-enabled multidimensional physical activity feedback in primary care patients at risk of chronic disease - the MIPACT study: a randomised controlled trial. International journal of behavioral nutrition and physical activity. 2020;17(1):99.
248.
Pelletier C, Chabot C, Rheaume C, Gagnon MP, Almeras N, Despres JP, et al. A randomized pilot trial: Using an activity tracker to increase motivation for physical activity in patients with type 2 diabetes in primary care. Diabetes Technol Ther. 2021;23(SUPPL 2):A170.
249.
Piette J, Valenstein M, Himle J, Duffy S, Torres T, Vogel M, et al. Clinical complexity and the effectiveness of an intervention for depressed diabetes patients. Chronic illness. 2011;7(4):267-278.
250.
Pinidiyapathirage J, Kasthuriratne A, Bennie JA, Pathmeswaran A, Biddle SJH, De Silva HJ, et al. Physical Activity Tracking Among Sri Lankan Adults: Findings From a 7-Year Follow-up of the Ragama Health Study. Asia-Pacific journal of public health. 2021;(asj, 8708538):1010539520971179.
251.
Pitta F, Troosters T, Spruit MA, Probst VS, Decramer M, Gosselink R. Characteristics of physical activities in daily life in chronic obstructive pulmonary disease. American Journal of Respiratory & Critical Care Medicine. 2005;171(9):972–7.
252.
Pitta F., Breyer M.-K., Hernandes N.A., Teixeira D., Sant'Anna T.J.P., Fontana A.D., et al. Comparison of daily physical activity between COPD patients from Central Europe and South America. Respiratory Medicine. 2009;103(3):421–6.
253.
Pizzol D., Smith L., Koyanagi A., Stubbs B., Grabovac I., Jackson S.E., et al. Do older people with diabetes meet the recommended weekly physical activity targets? An analysis of objective physical activity data. International Journal of Environmental Research and Public Health. 2019;16(14):2489.
254.
Pozehl BJ, Mcguire R, Duncan K, Hertzog M, Deka P, Norman J, et al. Accelerometer-Measured Daily Activity Levels and Related Factors in Patients With Heart Failure. Journal of Cardiovascular Nursing. 2018;33(4):329–35.
255.
Prieto-Centurion V., Casaburi R., Coultas D.B., Kansal M.M., Kitsiou S., Luo J.J., et al. Daily physical activity in patients with COPD after hospital discharge in a minority population. Chronic Obstructive Pulmonary Diseases. 2019;6(4):332–40.
256.
Qi Q., Wang X., Strizich G., Sotres-Alvarez D., Buelna C., Gallo L., et al. Associations of objectively-measured sedentary time and physical activity with meeting cardiovascular risk factor control goals in U.S. Hispanic/latino adults with diabetes: The hispanic community health study/study of latinos (HCHS/SOL). Circulation. 2016;133(SUPPL. 1).
257.
Ramakrishnan R., Doherty A., Smith-Byrne K., Rahimi K., Bennett D., Woodward M., et al. Accelerometer measured physical activity and the incidence of cardiovascular disease: Evidence from the UK Biobank Cohort study. PLoS Medicine. 2021;18(1):e1003487.
258.

Reddy YNV, Obokata M, Jones AD, Lewis GD, Shah SJ, Abouezzedine OF, et al. Characterization of the Progression From Ambulatory to Hospitalized Heart Failure With Preserved Ejection Fraction. Journal of Cardiac Failure. 2020;26(11):919–28.
259.
Reichmann W.M., Katz J.N., Burbine S.A., Daigle M.E., Rome B.N., Weinstein A.M., et al. Adherence to physical activity guidelines and its relationship with self-rated health among persons with doctor-diagnosed arthritis. Arthritis and Rheumatism. 2011;63(10 SUPPL. 1).
260.
Richardson CR, Goodrich DE, Larkin AR, Ronis D, Holleman RG, Damschroder LJ, et al. A Comparative Effectiveness Trial of Three Walking Self-monitoring Strategies. Translational journal of the American College of Sports Medicine. 2016;1(15):133–42.
261.
Rinaldo R.F., Parazzini E., Brambilla E., Longo P., Comandini S., Alfano F., et al. How does severe asthma impact on everyday life? European Respiratory Journal. 2019;54(Supplement 63).
262.
Rodrigues Bueno D, Nunes Marucci M de F, da Costa Rosa CS, Araújo Fernandes R, Aparecida de Oliveira Duarte Y, Lebão ML. Objectively Measured Physical Activity and Healthcare Expenditures Related to Arterial Hypertension and Diabetes Mellitus in Older Adults: SABE Study. Journal of Aging & Physical Activity. 2017;25(4):553–8.
263.
Rosenzweig E, Fatima K, Valencia G, Pierce V, Katiyal A, Rosenzweig E, et al. Does accelerometry provide more accurate real-time information to evaluate and follow-up patients with pulmonary arterial hypertension? Pulmonary circulation Conference: 11th international conference on neonatal and childhood pulmonary vascular disease United states. 2018;8(3):11-12.
264.
Rossen J., Buman M.P., Johansson U.-B., Yngve A., Ainsworth B., Brismar K., et al. Reallocating bouted sedentary time to non-bouted sedentary time, light activity and moderate-vigorous physical activity in adults with prediabetes and type 2 diabetes. PLoS ONE. 2017;12(7):e0181053.
265.
Sagawa N., Rockette-Wagner B., Azuma K., Ueshima H., Hisamatsu T., Takamiya T., et al. Physical activity levels in American and Japanese men from the ERA-JUMP Study and associations with metabolic syndrome. Journal of Sport and Health Science. 2020;9(2):170–8.
266.
Sandbakk S, Nauman J, Zisko N, Sandbakk O, Aspvik N, Stensvold D, et al. Sedentary Time, Cardiorespiratory Fitness, and Cardiovascular Risk Factor Clustering in Older Adults–the Generation 100 Study. Mayo clinic proceedings. 2016;91(11):1525-1534.
267.
Saner H, Schutz N, Botros A, Urwyler P, Buluschek P, du Pasquier G, et al. Potential of Ambient Sensor Systems for Early Detection of Health Problems in Older Adults. Frontiers in cardiovascular medicine. 2020;7(101653388):110.
268.
Sano Y., Matsuoka R., Sato Y., Kimura S., Mihara S., Kurosawa H. Pedometer intervention to improve daily physical activity and quality of life in patients with stable COPD. Chest. 2017;152(4 Supplement 1):A980.
269.

Sano Y., Matsuoka R., Sato Y., Kimura S., Mihara S., Kurosawa H. Effects of pedometer intervention on daily physical activity and qol in patients with COPD, including additional analysis on ACO. Respirology. 2018;23(Supplement 2):249–50.
270.
Sano Y., Matsuoka R., Sato Y., Kimura S., Mihara S., Kurosawa H. Pedometer intervention to improve daily physical activity and QOL in patients with stable COPD including those with ACO. European Respiratory Journal. 2018;52(Supplement 62).
271.
Sartini C., Wannamethee S.G., Iliffe S., Morris R.W., Ash S., Lennon L., et al. Diurnal patterns of objectively measured physical activity and sedentary behaviour in older men. BMC public health. 2015;15((Sartini, Wannamethee, Iliffe, Morris, Ash, Lennon, Jefferis) Department of Primary Care&Population Health, University College London, Rowland Hill Street, NW3 2PF, London, UK):609.
272.
Saxer S., Hasler E., Furian M., Bloch K., Ulrich S. Physical activity in COPD and its relationship to exercise performance and sleep. European Respiratory Journal. 2016;48(Supplement 60).
273.
Saxer S., Hasler E.D., Furian M., Bloch K.E., Ulrich S. Physical activity in COPD and its relationship to exercise performance and sleep. Kardiovaskulare Medizin. 2016;19(5 Supplement 26):88S.
274.
Schock D., Neher J.O., Safranek S., Kelsberg G. Q Do pedometers increase activity and improve health outcomes? Journal of Family Practice. 2017;66(1):48–9.
275.
Schüz N, Walters J, Cameron-Tucker H, Scott J, Wood-Baker R, Walters E. Patient Anxiety and Depression Moderate the Effects of Increased Self-management Knowledge on Physical Activity: a Secondary Analysis of a Randomised Controlled Trial on Health-Mentoring in COPD. COPD. 2015;12(5):502-509.
276.
Scott Kehler D., Horne D., Hiebert B., Kaoukis G., Garcia E., Babiak I., et al. Impact of physical activity on depression after cardiac surgery (IPAD-CS)-Preliminary results. Journal of Cardiopulmonary Rehabilitation and Prevention. 2011;31(5):E2.
277.
Scragg J., Okwose N., Cassidy S., MacGowan G., Bailey K., Skiner J., et al. Association between physical activity and cardiac performance in chronic heart failure. European Journal of Preventive Cardiology. 2017;24(1 Supplement 1):S100.
278.
Semanik P, Lee J, Manheim L, Dipietro L, Dunlop D, Chang R. Relationship between accelerometer-based measures of physical activity and the Yale Physical Activity Survey in adults with arthritis. Arthritis care & research. 2011;63(12):1766-1772.
279.
Semanik PA, Jungwha Lee, Jing Song, Chang RW, Min-Woong Sohn, Ehrlich-Jones LS, et al. Accelerometer-Monitored Sedentary Behavior and Observed Physical Function Loss. American Journal of Public Health. 2015;105(3):560–6.
280.
Shang-Lin C, Chien-Lung S, Liang-Cheng C, Yi-Pang L, Chueh-Ho L, Chia-Huei L. Effectiveness of a Home-Based Telehealth Exercise Training Program for Patients With

Cardiometabolic Multimorbidity: a Randomized Controlled Trial. Journal of cardiovascular nursing. 2020;35(5):491-501.
281.
Shiraishi M., Higashimoto Y., Maeda K., Okajima S., Sugiya R., Nishiyama O., et al. Ralationship between physical activity and depression in patients with COPD participating in an outpatient pulmonary rehabilitation program. American Journal of Respiratory and Critical Care Medicine. 2015;191(MeetingAbstracts).
282.
Siddiqui M.A., Bhana S., Daya R. The relationship between objectively measured physical activity and parameters of disease control in an African population of type 2 diabetes mellitus. Journal of Endocrinology, Metabolism and Diabetes of South Africa. 2018;23(3):80–5.
283.
Skotzko C.E., Krichten C., Zietowski G., Alves L., Freudenberger R., Robinson S., et al. Depression is common and precludes accurate assessment of functional status in elderly patients with congestive heart failure. Journal of Cardiac Failure. 2000;6(4):300–5.
284.
Smith KJ, Pedneault M, Schmitz N. Investigation of anxiety and depression symptom co-morbidity in a community sample with type 2 diabetes: Associations with indicators of self-care. Canadian journal of public health = Revue canadienne de sante publique. 2016;106(8):e496-501.
285.
Smuck M., Kao M.-C., Goldin M., Patel A. The association of accelerometer-based activity monitoring with chronic low back pain. Spine Journal. 2011;11(10 SUPPL. 1):89S.
286.
Snipelisky D, Kelly J, Levine J, Koepp G, Anstrom K, McNulty S, et al. Accelerometer-Measured Daily Activity in Heart Failure With Preserved Ejection Fraction: clinical Correlates and Association With Standard Heart Failure Severity Indices. Circulation Heart failure. 2017;10(6):e003878.
287.
Snipelisky D., Kelly J., Levine J.A., Koepp G.A., Anstrom K.J., McNulty S.E., et al. Accelerometer measured daily activity in heart failure with preserved ejection fraction: Clinical correlates and association with standard heart failure severity indices. Circulation. 2016;134(Supplement 1).
288.
Song J., Dunlop D.D., Semanik P., Chang A.H., Jackson R.D., Chang R.W., et al. Reallocating time spent in sleep, sedentary behavior and physical activity and its association with pain and depression. Arthritis and Rheumatology. 2017;69(Supplement 10).
289.
Soo Kyung Park, Larson JL. The Relationship Between Physical Activity and Metabolic Syndrome in People With Chronic Obstructive Pulmonary Disease. Journal of Cardiovascular Nursing. 2014;29(6):499–507.
290.
Sperb L.F., De Paula T.P., Moreira J.S.R., De Freitas M.M., Miller M.E.P., Da Silva A.S., et al. Vitamin D deficiency is associated with high blood pressure in 24-h ambulatory blood pressure monitoring in patients with type 2 diabetes. Endocrine Reviews. 2017;38(3 Supplement 1).
291.
Steele B, Dougherty C, Burr R, Gylys-Colwell I, Hunziker J. An intervention to enhance function in severe cardiopulmonary illness. American journal of respiratory and critical care medicine. 2012;185.

292.
Steeves J.A., Shiroma E.J., Conger S.A., Van Domelen D., Harris T.B. Physical activity patterns and multimorbidity burden of older adults with different levels of functional status: NHANES 2003-2006. Disability and Health Journal. 2019;12(3):495–502.
293.
Stuart T., Rogers C., Balanos G., Wood A.M. The relationship of home activity levels to psychological Co-morbidity in COPD. Thorax. 2011;66(SUPPL. 4):A85.
294.
Swartz A.M., Strath S.J., Parker S.J., Miller N.E. The impact of body-mass index and steps per day on blood pressure and fasting glucose in older adults. Journal of Aging and Physical Activity. 2008;16(2):188–200.
295.
Takae R., Hatamoto Y., Yasukata J., Kose Y., Komiyama T., Ikenaga M., et al. Physical activity and/or high protein intake maintains fat-free mass in older people with mild disability; the fukuoka island city study: A cross-sectional study. Nutrients. 2019;11(11):2595.
296.
Takahashi P, Quigg S, Croghan I, Schroeder D, Ebbert J. Effect of pedometer use and goal setting on walking and functional status in overweight adults with multimorbidity: a crossover clinical trial. Clinical interventions in aging. 2016;11:1099-1106.
297.
Takahashi T., Kumamaru M., Morisawa T., Yamada S., Matsuda H. Does amount of physical activity relate to functional and psychological recovery after cardiac surgery? Physiotherapy (United Kingdom). 2011;97(SUPPL. 1):eS1206.
298.
Tamaki A., Kawaura G., Fujisawa C., Ikushima H. Relationship between physical activity, muscle thickness, and echo intensity in patients with chronic obstructive pulmonary disease: A cross-sectional study. European Respiratory Journal. 2017;50(Supplement 61).
299.
Tanaka S., Fujita K., Makimoto K., Kanaoka M., Yakushiji K., Tanaka R., et al. Relationships of accelerometer-determined physical activity with obesity, hypertension, diabetes, dyslipidemia, and health-related quality of life in patients after liver transplantation. Clinical Transplantation. 2020;34(12):e14117.
300.
Taylor A, Taylor R, Ingram W, Dean S, Jolly K, Mutrie N, et al. Randomised controlled trial of an augmented exercise referral scheme using web-based behavioural support for inactive adults with chronic health conditions: the e-coachER trial. British journal of sports medicine. 2020;
301.
Taylor A, Taylor R, Ingram W, Dean S, Jolly K, Mutrie N, et al. Randomised controlled trial of an augmented exercise referral scheme using web-based behavioural support for inactive adults with chronic health conditions: the e-coachER trial. British journal of sports medicine. 2021;55(8):444–50.
302.
Thakkar N., Jamnik V., Ardern C.I. Cross-associations between physical activity and sedentary time on metabolic health: a comparative assessment using self-reported and objectively measured activity. Journal of public health (Oxford, England). 2018;40(4):e464–73.
303.

Thanoo N., Gilbert A., Song J., Dunlop D.D., Chang R.W. The relationship between the number of prescription medications and physical activity amongst patients with or at high risk for knee osteoarthritis. Arthritis and Rheumatology. 2017;69(Supplement 10).
304.
Thanoo N, Gilbert AL, Trainor S, Semanik PA, Song J, Lee J, et al. The Relationship between Polypharmacy and Physical Activity in Those with or at Risk of Knee Osteoarthritis. Journal of the American Geriatrics Society. 2020;68(9):2015–20.
305.
Tomkins-Lane C, Lafave L, Parnell J, Rempel J, Moriartey S, Andreas Y, et al. The spinal stenosis pedometer and nutrition lifestyle intervention (SSPANLI): development and pilot. Spine journal. 2015;15(4):577-586.
306.
Troosters T., Sciurba F., Battaglia S., Langer D., Valluri S.R., Martino L., et al. Physical inactivity in patients with COPD, a controlled multi-center pilot-study. Respiratory Medicine. 2010;104(7):1005–11.
307.
Tudor-Locke C., Schuna J.M., Han H.O., Aguiar E.J., Green M.A., Busa M.A., et al. Step-Based Physical Activity Metrics and Cardiometabolic Risk: NHANES 2005-2006. Medicine and science in sports and exercise. 2017;49(2):283–91.
308.
Tudor-Locke C.E., Bell R.C., Myers A.M., Harris S.B., Lauzon N., Rodger N.W. Pedometer-determined ambulatory activity in individuals with type 2 diabetes. Diabetes Research and Clinical Practice. 2002;55(3):191–9.
309.
Ullrich P, Werner C, Bongartz M, Kiss R, Bauer J, Hauer K. Validation of a Modified Life-Space Assessment in Multimorbid Older Persons With Cognitive Impairment. Gerontologist. 2019;59(2):e66-e75.
310.
Uritani D, Hinman R, Kasza J, Campbell P, Egerton T, Metcalf B, et al. The association between physical activity and psychological characteristics in people with knee osteoarthritis. Osteoarthritis and cartilage. 2018;26:S350-.
311.
Vaidya T., Badatcheff C., Bellocq A., Costes F., Ouksel H., De Bisschop C., et al. Physical activity evolution measured by actimeter in COPD patients after a pulmonary rehabilitation. European Respiratory Journal. 2018;52(Supplement 62).
312.
van den Berg-Emons RJ, Bussmann JB, Stam HJ. Accelerometry-Based Activity Spectrum in Persons With Chronic Physical Conditions. Archives of Physical Medicine & Rehabilitation. 2010;91(12):1856–61.
313.
Van Der Berg J.D., Stehouwer C.D.A., Bosma H., Van Der Velde J.H.P., Willems P.J.B., Savelberg H.H.C., et al. Objectively measured sedentary behaviour patterns according to diabetes status: The Maastricht Study. Diabetologia. 2015;58(1 SUPPL. 1):S95–6.
314.
van der Zee-Neuen A., Wirth W., Osterbrink J., Hosl K., Eckstein F. The association of physical activity and depression in patients with or at risk of osteoarthritis is captured equally well by patient reported outcomes and accelerometers. Osteoarthritis and Cartilage. 2019;27(Supplement 1):S246–7.

315.
Van Remoortel H., Hornikx M., Saucedo Marquez C.M., Avila A., Burtin C., Langer D., et al. The impact of mild COPD on daily physical activity in patients with overweight. American Journal of Respiratory and Critical Care Medicine. 2011;183(1 MeetingAbstracts).
316.
Van Remoortel H, Hornikx M, Langer D, Burtin C, Everaerts S, Verhamme P, et al. Risk factors and comorbidities in the preclinical stages of chronic obstructive pulmonary disease. American Journal of Respiratory & Critical Care Medicine. 2014;189(1):30–8.
317.
Vasquez P.M., Durazo-Arvizu R.A., Marquez D.X., Argos M., Lamar M., Odoms-Young A., et al. Physical activity, stress, and cardiovascular disease risk: HCHS/SOL Sociocultural Ancillary Study. Preventive Medicine Reports. 2020;20((Vasquez) Department of Urban Public Health, Charles R. Drew University of Medicine and Science, Los Angeles, CA, United States):101190.
318.
Vileikyte L., Jiun Shen B., Brown S., Boulton A.J.M., Kirsner R., Reeves N., et al. Depression, physical activity, and diabetic foot ulcer healing. Diabetes. 2017;66(Supplement 1):A168.
319.
Walters J., Wills K., Nelson M.R., Robinson A., Turner P., Scott J., et al. Free living physical activity in community-based patients with moderate or severe copd assessed by accelerometer. Respirology. 2011;16(SUPPL. 1):23.
320.
Walters J, Wills K, Robinson A, Nelson M, Scott J, Turner P, et al. Effect of health-mentoring to increase daily physical activity in chronic obstructive pulmonary disease (COPD). Respirology (carlton, vic). 2012;17:61.
321.
Wan E, Kantorowski A, Teylan M, Kadri R, Richardson C, Garshick E, et al. Patterns of change in daily step count among COPD patients enrolled in A 3-month physical activity intervention. American journal of respiratory and critical care medicine. 2017;195.
322.
Waschki B., Spruit M.A., Watz H., Albert P., Shrikrishnapalasuriyar D., Smith C., et al. Measurement of physical activity in patients with COPD in a multicenter setting: Compliance and associations with clinical characteristics of the disease. American Journal of Respiratory and Critical Care Medicine. 2011;183(1 MeetingAbstracts).
323.
Waschki B, Kirsten AM, Holz O, Mueller KC, Schaper M, Sack AL, et al. Disease Progression and Changes in Physical Activity in Patients with Chronic Obstructive Pulmonary Disease. American Journal of Respiratory & Critical Care Medicine. 2015;192(3):295–306.
324.
Weinstock R, Brooks G, Palmas W, Morin P, Teresi J, Eimicke J, et al. Lessened decline in physical activity and impairment of older adults with diabetes with telemedicine and pedometer use: results from the IDEATel study. Age and ageing. 2011;40(1):98-105.
325.
Wendt A, Wehrmeister FC, Ricardo LIC, Silva BGC da, Martins RC, Gonçalves H, et al. Objectively measured physical activity according to the periods of the day in the Pelotas Cohort. Revista Brasileira de Atividade Física & Saúde. 2020 Dec 10;25:1–10.
326.
Westbury LD, Dodds RM, Syddall HE, Baczynska AM, Shaw SC, Dennison EM, et al. Associations Between Objectively Measured Physical Activity, Body Composition and

Sarcopenia: Findings from the Hertfordshire Sarcopenia Study (HSS). Calcif Tissue Int. 2018 Sep 1;103(3):237–45.
327.
Whipple M.O., Schorr E., Treat-Jacobson D. Patterns of sedentary behavior on exercise and non-exercise days among older adults referred for supervised exercise therapy for peripheral artery disease. Circulation. 2018;138(Supplement 1).
328.
Whipple MO, Schorr EN, Talley KMC, Lindquist R, Bronas UG, Treat-Jacobson D. A mixed methods study of perceived barriers to physical activity, geriatric syndromes, and physical activity levels among older adults with peripheral artery disease and diabetes. Journal of Vascular Nursing. 2019;37(2):91–105.
329.
White D.K., Felson D.T., Zhang Y., Gross K.D., Niu J., Nevitt M.C., et al. Is knee pain relevant for meeting physical activity guidelines among people with knee osteoarthritis who can walk already walk at a moderate intensity? The multicenter osteoarthritis study. Arthritis and Rheumatism. 2011;63(10 SUPPL. 1).
330.
Witham M, Argo I, Johnston D, Struthers A, McMurdo M. Long-term follow-up of very old heart failure patients enrolled in a trial of exercise training. American journal of geriatric cardiology. 2007;16(4):243-248.
331.
Woolf K., Reese C.E., Mason M.P., Beaird L.C., Tudor-Locke C., Vaughan L.A. Physical Activity Is Associated with Risk Factors for Chronic Disease across Adult Women's Life Cycle. Journal of the American Dietetic Association. 2008;108(6):948–59.
332.
Xavier R., Lopes A.C., Pereira A.C.A.C., Carvalho-Pinto R.M., Fernandes F.L.A., Harrison S.L., et al. Association of illness perceptions with psychodemographic factors and physical activity in COPD patients. European Respiratory Journal. 2016;48(Supplement 60).
333.
Xavier R., Lopes A.C., Pereira A.C.A.C., Mancini M.C., Ramos E.M.C., Stelmach R., et al. Factors associated with daily life physical activity in Brazilian COPD patients. European Respiratory Journal. 2016;48(Supplement 60).
334.
Xavier R., Pereira A.C.A.C., Lopes A.C., Ramos E.M.C., Mancini M.C., Cukier A., et al. Comparison of daily life physical activity in patients with COPD and asthma COPD overlap syndrome. European Respiratory Journal. 2016;48(Supplement 60).
335.
Yang A, Makris U, Spence N, Saxon L, LePage J, Reid MC, et al. Improving outcomes in older veterans with chronic low back pain and comorbid depression: Preliminary data from the motivate pilot trial. J Am Geriatr Soc. 2021;69(SUPPL 1):S131–2.
336.
Yang G.-R., Dye T.D., Li D. Association between diabetes, metabolic syndrome and heart attack in US adults: A cross-sectional analysis using the Behavioral Risk Factor Surveillance System 2015. BMJ Open. 2019;9(9):e022990.
337.
Yates T, Haffner S, Schulte P, Thomas L, Huffman K, Bales C, et al. Association between change in daily ambulatory activity and cardiovascular events in people with impaired glucose tolerance (NAVIGATOR trial): a cohort analysis. Lancet (london, england). 2014;383(9922):1059-1066.

338.
Yates TE. Can we out-walk the type 2 diabetes mellitus epidemic? [Internet]. 2008 [cited 2021 Mar 1]. Available from: http://hdl.handle.net/10068/958072
339.
Yoshida C., Ichiyasu H., Ideguchi H., Hirosako S., Masunaga A., Kojima K., et al. Four-meter gait speed predicts daily physical activity in patients with chronic respiratory diseases. Respiratory Investigation. 2019;57(4):368–75.
340.
Yoshiuchi K., Nakahara R., Kumano H., Kuboki T., Togo F., Watanabe E., et al. Yearlong physical activity and depressive symptoms in older Japanese adults: Cross-sectional data from the Nakanojo study. American Journal of Geriatric Psychiatry. 2006;14(7):621–4.
341.
Zhang Q, Schwade M, Schafer P, Weintraub N, Young L. Characterization of Sedentary Behavior in Heart Failure Patients With Arthritis. Cardiology research. 2020;11(2):97-105.
342.
Zhu Z, Han W, Wang K, Cicuttini F, Ding C, Jones G. The relationship between physical activity and MRI-detected osteophyte is modified by common risk factors for osteoarthritis. Osteoporosis international. 2018;29(1):S152-.
343.
Zisko N., Nauman J., Sandbakk S.B., Aspvik N.P., Salvesen O., Carlsen T., et al. Absolute and relative accelerometer thresholds for determining the association between physical activity and metabolic syndrome in the older adults: The Generation-100 study. BMC geriatrics. 2017;17(1):109.
344.
Association of the change in physical activity and cardiovascular disease outcomes in the look ahead trial. Circulation. 2018;Conference: American Heart Association's Epidemiology and Prevention/Lifestyle and Cardiometabolic Health 2018. United States. 137(Supplement 1).
345.
Associations of objectively measured moderate-to-vigorous-intensity physical activity and sedentary time with all-cause mortality in a population of adults at high risk of type 2 diabetes mellitus | arc-em.nihr.ac.uk [Internet]. [cited 2021 Mar 1]. Available from: https://arc-em.nihr.ac.uk/resources/associations-objectively-measured-moderate-vigorous-intensity-physical-activity-and
