# Supplementary figures and images for: Objectively measured physical activity levels and adherence to physical activity guidelines in people with multimorbidity—A systematic review and meta-analysis
Source: PLoS One. 2022 Oct 12;17(10):e0274846. doi: 10.1371/journal.pone.0274846 (PMC9555650; doi:10.1371/journal.pone.0274846)

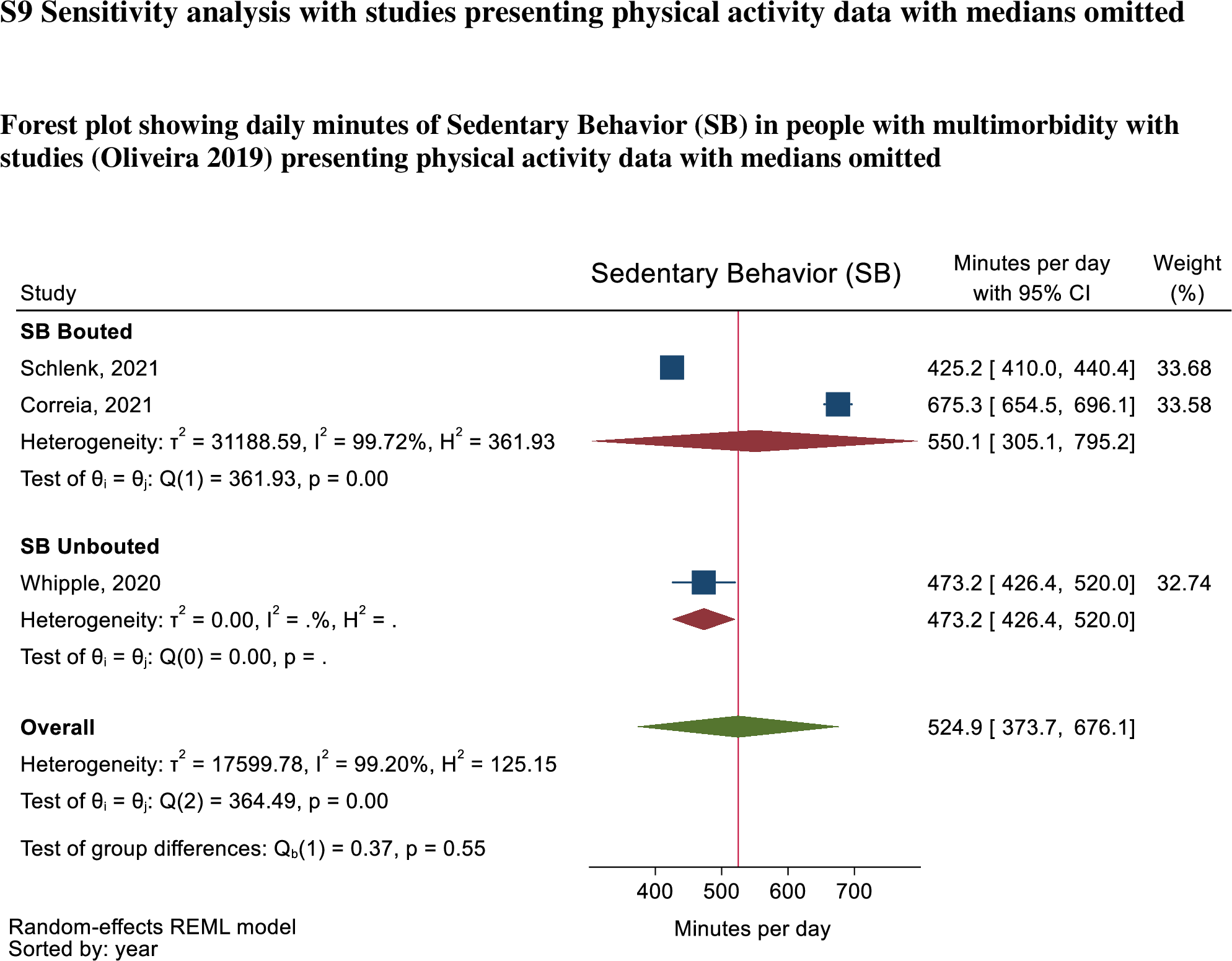

Supplement: S1 Fig — (TIF) [file pone.0274846.s009.tif]

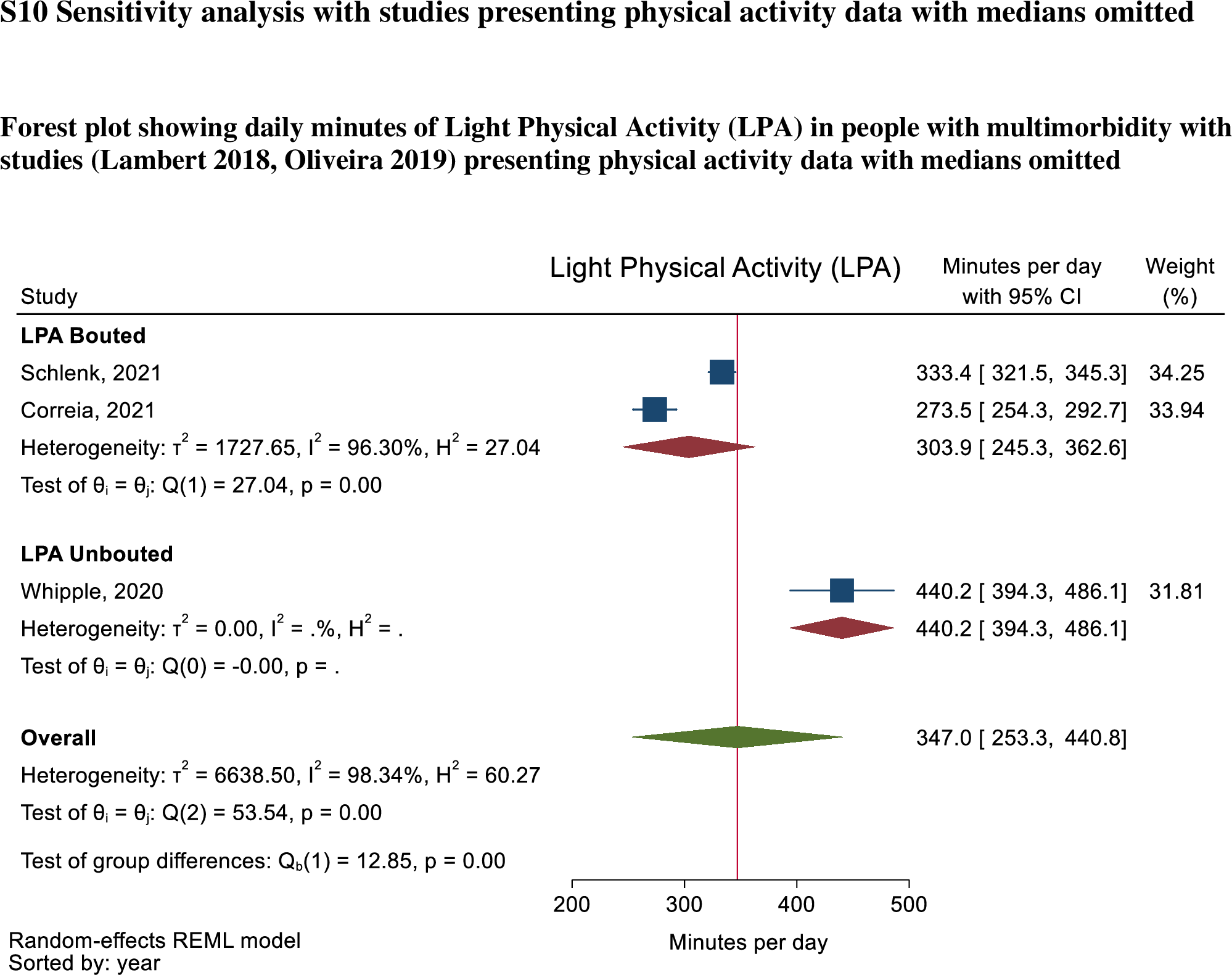

Supplement: S2 Fig — (TIF) [file pone.0274846.s010.tif]

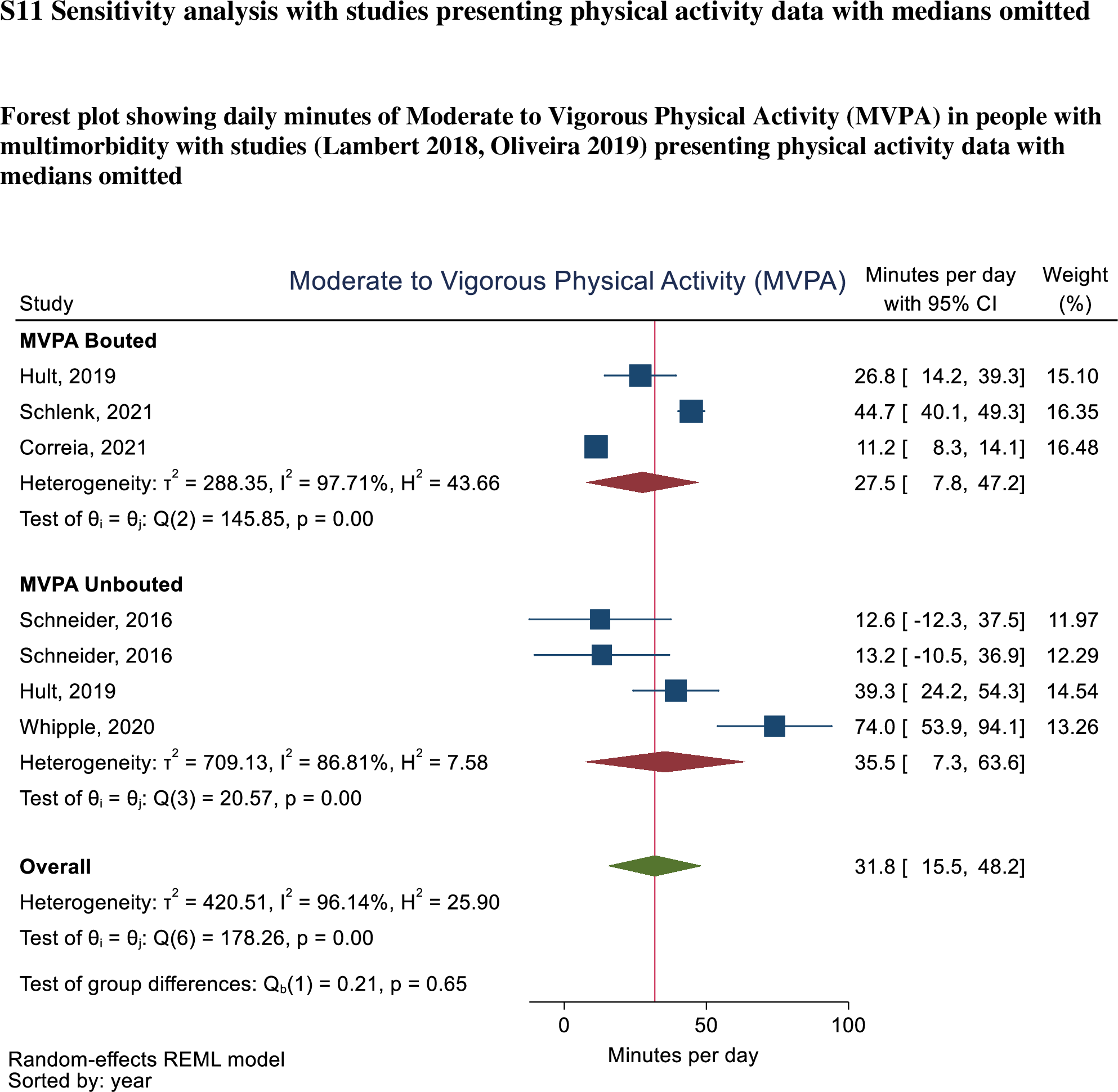

Supplement: S3 Fig — (TIF) [file pone.0274846.s011.tif]
