# Supplementary material for: Objectively measured physical activity levels and adherence to physical activity guidelines in people with multimorbidity—A systematic review and meta-analysis
Source: PLoS One. 2022 Oct 12;17(10):e0274846. doi: 10.1371/journal.pone.0274846 (PMC9555650; doi:10.1371/journal.pone.0274846)
Supplement: Attachment — Submitted filename: Response to Reviewers.docx [file pone.0274846.s013.docx]

Dear Editor - Dr. Rafael Franco Soares Oliveira,

We wish to thank both the academic editor and reviewers for their relevant comments on our manuscript; *‘Objectively measured physical activity levels and adherence to physical activity guidelines in people with multimorbidity: a systematic review and meta-analysis’* (PONE-D-22-18651). We have carefully read and provided responses to address all the comments and concerns raised in the peer review and hereby submit a revised version of our paper.

Our detailed point-by-point responses are provided below and all changes are marked in the revised manuscript as requested. We have submitted two versions of the manuscript as requested, one with and another without changes tracked named 'Revised Manuscript with Track Changes' and ‘Manuscript’, respectively - page and line numbers in the response to reviewers are referring to the version of the manuscript with track changes.

Besides increasing the quality and clarity of our paper, we hope that the changes made to the manuscript now makes it suitable for publication in PLOS ONE. We look very much forward to receiving your response to our resubmitted paper.

On behalf of all authors,

Lars Bo Jørgensen, PhD fellow

Department of Sports Science and Clinical Biomechanics

University of Southern Denmark

| **Comment** | **Authors reply** | **Action taken / Manuscript revisions** |
| --- | --- | --- |
| **Editor** |  |  |
| -check the numerical order of the sections of your work (e.g. introduction should be 1 and methods should be 2, and so on); |  | The numerical order has been edited as requested. |
| -section/name "protocol" does exist in PRISMA 2020, please check; | The authors fully acknowledge that following the specific order of the PRISMA 2020 guidelines in general eases the readability of the paper and thank the editor for this comment. | A section entitled ‘Other information’ was added to the paper *(lines 447-449).* Text regarding the review protocol was moved to this section and given a separate sub-heading (‘5.1 Registration and protocol’) as suggested in the PRISMA 2020 guidelines. The remaining sentences of the paragraph in the methods section was changed from:  *‘The review protocol was registered at PROSPERO (CRD42020172456) and followed the recommendations for performing systematic reviews as described in the Cochrane Handbook’*  to;  *‘This review followed the recommendations for performing systematic reviews as described by Cochrane…’* |
| - before synthesis of methods, please add "effect measures"; | The authors thank the editor for this suggestion, which has been implemented in the manuscript. | A section on effect measures was added to the manuscript  *2.8 Effect measures*  *2.8.1 Estimate measures*  *Average minutes of daily activity spent as SB, LPA, MPA, MVPA, VPA and steps was assessed in separate meta-analyses. (lines 205-208)* |
| - after synthesis of methods, please add "Reporting bias assessment" | The authors thank the editor for this suggestion, which we have further clarified in the manuscript using a stand-alone paragraph. | A section on reporting bias assessment was added to the manuscript.  *2.9.3 Reporting bias assessment*  *Authors of potentially eligible studies were contacted about outcome and population characteristics. For example, asking for measure of variance (e.g. standard deviation) regarding PA measures and prevalence of chronic conditions in the reported population when it was only reported that the population had multimorbidity. All contact to author was done by e-mail to ensure the greatest response rate possible [32]. Initially an e-mail was sent to the corresponding author. If an answer was not received within fifteen workdays, the last author was contacted, and the deadline extended with fifteen more days. If no reply was received, the study was excluded (lines 235-242)* |
| **Reviewer #1 (Dr. Christophe Domingos)** |  |  |
| Lines 157 to 158: it is said that the authors considered medians equivalent to the mean. Could you explain it better, knowing that the variables are different and have different meanings? How could the outcome be different if the variables were the same? | We thank the reviewer for the comment and agree that additional information will benefit the clarity and quality of the manuscript.  In the previous manuscript, and in line with the Cochrane Handbook recommendation, we pooled mean and median values in meta-analysis, given that median values can be very similar to mean values when the distribution of the data is not skewed. Since we were unware of the distribution of the original variables, we have performed an additional sensitivity analysis removing studies reporting median values *(Lambert et al. 2018 og Oliveira et al. 2019*) and added this analysis as supplementary information. Importantly, results from the additional analysis were in line with the results originally presented. | Further analysis was added to the manuscript and described under section 2.9.4 *(lines 244-247)* and section 3.3.1 *(lines 309-310).*  *2.9.4 Sensitivity analysis*  *Sensitivity analysis was performed by removing studies presenting median values to check the robustness of the findings, give that mean and median values were pooled in the same meta-analyses as recommended by Cochrane [23].*  And  *The sensitivity analysis removing studies reporting median values displayed similar results as the main analyses (S9).* |
| Lines 171 to 172: Instead of "questions not considered relevant," consider replacing it with "questions which were not the same as the aim of the review." If the authors use the first one, readers will never know what questions were not relevant or considered irrelevant by the authors. | We would like to thank the reviewer for this comment and agree that the transparency of the development of the modified assessment tool could be increased by elaborating this section of the manuscript. We have therefore added text to the manuscript to exemplify the modifications made to the assessment tool. | *‘Questions not considered relevant for this review were removed and options to answer, and wording altered to fit the objectives of the review’*  Was changed to;  *‘Questions not considered relevant for the aims of this review were deleted. The deleted questions were: Rating of study design, Confounders, Withdrawals and drop-outs, Intervention integrity and Analyses. Furthermore, options to answer, and wording was altered to fit the aims of the review e.g. ‘Are the individuals selected to participate in the study likely to be representative of the target population?’ was replaced with ‘How representative was the study participants of the multimorbid target population?’ (lines 186-191)* |
| Lines 198: It could be better to replace "we performed" for "a narrative synthesis of the results was performed.". |  | Edited according to reviewers suggestion *(line 226)* |
| Line 217: Please, replace "was" for "were". |  | Edited according to reviewers suggestion *(line 259)* |
| Line 229: Consider the following change - "Fifteen studies were included...". |  | Edited according to reviewers suggestion *(line 272)* |
| Lines 284; It could be interesting to add - "% gender and different ethnicities since they are variables known for their massive impact on PA and morbidities." | We thank the reviewer for drawing our attention to this relevant aspect. We were unfortunately not able to perform additional analyses on several variables that are known to have an impact on PA and morbidities, e.g. gender ethnicities and socioeconomic status. We have implemented the reviewers comment by adding elaborating text to the manuscript. | Text wad added;  *Additional analyses on the impact of gender, ethnicity or socioeconomic status could not be performed since too few studies reported these variables consistently (line 417)* |
| Line 295: Typo "conditionswith". |  | Edited according to reviewers suggestion *(line 409)* |
| Line 314: Consider replacing "our meta-analysis" with "this meta-analysis.". |  | Edited according to reviewers suggested *(line 341)* |
| Line 323: Same as above. |  | Edited according to reviewers suggestion *(line 360)* |
| Lines 335 to 337: May I suggest incentive PA from youth as a preventive measure of multimorbidity instead of promoting while they already are in trouble? It is also necessary at that stage, but it would be better to reinforce the needing to do PA even if people are healthy and not only when they already present symptoms. | We thank the reviewer for commenting on this highly relevant aspect. Authors agree that *primary* prevention of single or multiple chronic condition(s) are important, and have implemented the comment by adding text and further references *(Koolhaus et al 2017, Lounassalo et al. 2019 and Skou et al 2022)* to this section of the manuscript. | The text now reads;  *These benefits could potentially be even more important in older populations, since studies have shown that older people spent the vast majority of their time in SB [65]. Furthermore, current evidence also supports that the level of PA decreases with increasing age in both healthy [66] and multimorbid populations [18]. Despite an extensive knowledge gap in the area, clinicians should therefore promote PA for people with chronic conditions and multimorbidity given the documented health benefits [10,11]. However, they should also acknowledge the importance of primary prevention, since physical inactivity is a risk factor for development of chronic conditions and multimorbidity [8,67]. In populations failing to adhere to PA guidelines, special attention should be given to the importance of replacing SB with PA of any intensity level as recommended by the WHO [13].*  *(lines 388-396)* |
| Line 344: It could be better to replace - "In the present review". |  | Edited according to reviewers suggestion *(line 346)* |
| Line 349: Same as above or similar. It is believed that this review (..). |  | Edited according to reviewers suggestion *(line 350)* |
| Lines 353 to 354: Consider replacing for "(...) interpretation, only a few relevant studies were identified (...)". |  | Edited according to reviewers suggestion  *‘All meta-analyses demonstrated high statistical heterogeneity among the included studies. Results should therefore be interpreted with caution. Furthermore, adding to a cautious interpretation we only identified few relevant studies, all associated with a high risk of bias, leading to an overall certainty in the evidence that was very low. It is highly likely that future studies will change the PA estimates identified in our study.’*  Was changed to:  *‘All meta-analyses demonstrated high statistical heterogeneity among the included studies. Furthermore, only few relevant studies were identified, all associated with a high risk of bias, leading to an overall certainty in the evidence that was very low. The results should therefore be interpreted with caution. It is highly likely that future studies will change the PA estimates identified in this review’ (lines 411-420)* |
| 369: Consider the following - "The present review or similar.". |  | Edited according to reviewers suggestion *(line 436)* |
| 370: Consider the following - "It was found that". |  | Edited according to reviewers suggestion *(line 436)* |
| 377: Consider the following - "The review". |  | Edited according to reviewers suggestion *(line 443*) |
| Obviously, for this paper, it cannot be done, but maybe consider adding the time of practice as a factor in future reviews. | Authors agree that time of practice could be an important factor. |  |
| Remember that pedometers and accelerometers can be tricked (maybe consider it a limitation). | Authors thank the reviewer for this comment, and acknowledges that objective measurement methods in general have several limitations – ‘tricking’ being one of them. We have elaborated this by adding a reference *(Strath et al. 2013)* and text. | *No perfect tool exists for measuring PA, it is however suggested that researchers should incorporate appropriate objective measures specific to the PA behaviour of interest when examining PA in adults in free-living conditions [18].*  Was changed to;  *No perfect tool exists for measuring PA, and also objective measurement methods have displayed limitations [69]. It is however suggested that researchers should incorporate appropriate objective measures specific to the PA behavior of interest when examining PA in adults in free-living conditions [19]. (lines 426-428)* |
| Please, add DOI in the references. |  | Edited according to the reviewers suggestion. |
| **Reviewer #2 (unknown)** |  |  |
| Introduction: I believe that the relevance of the study is not properly justified. There is no information that shows whether or not this population practices a recommended amount of physical activity. We assume not, but that information does not exist. There's just this: "Physical inactivity represents an important target in the growing burden of multimorbidity, as it increases the risk of worsening health, development of other chronic conditions (8) and death (9)". Therefore, the objective of the study is not properly substantiated. | The authors wish to thank the reviewer for this comment. The authors agree that elaborating more on the evidence on the current level of adherence to physical activity recommendations in people with multimorbidity could further justify the relevance of our study. The reviewer’s comment has therefore been accommodated by adding text to the introduction.  As argued in the introduction *(lines 83-85)* and elaborated in the discussion *(lines 422-423)* - objective measurement methods have demonstrated higher accuracy than subjective methods. Researchers are therefore suggested to incorporate them in examinations of physical activity in adults in free-living conditions. Investigations of the level of physical activity in adults in free-living conditions was the focus of this review.  There has been a considerable increase in studies on physical activity and multimorbidity *(Xu, Xiaolin et al. 2017, Journal of global health)* as well as an increase in the use of objective measurement methods *(Silfee et al. 2018, Prev Med Rep) in* the past decade(s). Combined with emerging evidence supporting that also lower levels of physical activity are positively associated with health benefits *(Ekelund et al. 2019, BMJ)*, highlights the importance of a **detailed** overview of the level of physical activity also in populations with multimorbidity. Such overview, however challenged by the quality of existing evidence it might be, is provided in this review and meta-analysis. Adding to the already existing justification for the study, we believe that the introduction now justifies the relevance and importance of the study. We believe the overview of evidence presented in this review can be considered important to both fellow researchers and health professionals worldwide. | The two paragraphs in the introduction section now reads;  *The* *World Health Organization (WHO) recommends that adults with chronic conditions should perform at least 150–300 minutes of moderate-intensity aerobic PA, 75 to 150 minutes of vigorous-intensity PA (or an equivalent combination) per week [13]. However, despite the well-documented benefits of PA, many people do not adhere to PA guidelines [14]. This proportion increases with the number of chronic conditions i.e. the level of PA is lower among people with multimorbidity [15–17]. A recent study found that nearly 68% of older adults with multimorbidity fail to meet the level of PA recommended by the WHO [18].*  *Unfortunately, and in spite of emerging evidence there is still limited knowledge on PA level in people with multimorbidity. Furthermore, no overview of the level of objectively measured PA and adherence to PA guidelines is available, although objectively measured PA is considered more accurate and less susceptible to bias than self-reported PA [19–22]. Therefore, this systematic review and meta-analysis aimed to determine 1) the level of objectively measured PA in people with multimorbidity and 2) the proportion that adheres to the WHO PA guidelines for people with chronic conditions. (lines 74-88)* |
| Methods: The Kappa index should be used to identify the concordance level between reviewers. | The authors thank the reviewer for the comment. However, the Cochrane Handbook states that it is not recommend using kappa statistics since it is unlikely to convey the real impact of any disagreements in a review (*Cochrane Handbook 7.2.6, Introductory text*). Instead, it is recommended that the reasons for any disagreement be explored since they may reveal the need to revisit eligibility criteria or coding schemes for data collection. In this case, resulting changes should be reported. In the present study, no adjustments or changes to coding schemes or eligibility criteria was performed and therefore we did not describe it. We have however elaborated this section by adding to the text. | The text now reads;  *The extraction procedure was pilot tested on three studies and data compared among the reviewers in order to implement any adjustments to the data extraction form. No adjustments were made. (lines 140-142)* |
| Why ten years as the temporal window for search isn’t fully justified. | Authors would like to accommodate the reviewers comment. However, we did not limit the search to any shorter periods of time in this study. As described in section 2.2 *(line 117)* literature was searched from inception up until January 2022. |  |
| Why the option for RCT and observational studies? / Why other methodologies aren’t included? | This study aimed to investigate the proportion that adhered to WHOs recommendation for physical activity and the overall level of physical activity at different intensities (from sedentary behavior to vigorous activity) and steps in people with multimorbidity. Studies with other methodologies, such as mixed-methods or case-reports/case-series could therefore have been included, as indicated by the reviewers comment. The authors agree that the text can seem ambiguous and have therefore changed the wording of the text in section 2.1.1. so it better reflects that other study designs could have been included. However, and importantly, no other study designs than clinical trials (randomized and non-randomized) and observational studies were identified in the search, and only one study was excluded from the full text screening due to ‘wrong study design’ (a review). | *Clinical trials, randomized controlled trials (RCT), and observational studies where PA was measured objectively (e.g., activity monitor such as a pedometer or accelerometer) in an adult multimorbid population (mean age ≥18 years) were included.*  *From clinical and randomized controlled trials, we extracted baseline PA data.*  Was changed to;  *Studies where PA was measured objectively (e.g., activity monitor such as a pedometer or accelerometer) in an adult multimorbid population (mean age ≥18 years) were included. Only baseline data on PA was extracted. (lines 99-102)*  and the abstract now read;  *All studies, where PA was measured at baseline using an activity monitor in an adult (≥18 years) multimorbid (≥80% of the population had ≥2 chronic conditions) population. (lines 31-33)* |
| Why the option for those databases? | The authors acknowledge that other databases could have been searched. We did, however, follow the recommendations provided by the Cochrane Handbook *(Cochrane Handbook, 4.3.1)* and included both Medline, EMBASE and CENTRAL among the databases. It is the author’s belief that the combination of these databases along with the other information sources used in this review can be considered sufficient. |  |
| The subgroup analyses should be justified prior. | We thank the reviewer for drawing our attention to this and have further justified the subgroup-analyses by adding text and three references *(Loprinzi 2015, Wolff-Hughes 2015, Sagelv 2019)* to the section of the manuscript.  Furthermore, we added text to the Data Items section 2.6, to clarify the procedures used when including multiple groups from one study and to clarify that this was performed according to recommendations described in the Cochrane handbook *(Cochrane handbook section 16.5.4)*. | The following text;  *Meta-analyses were performed for minutes of activity spent as SB, LPA, MPA, MVPA, VPA and daily steps using a random-effects model given the heterogeneous population included and presented in forest plots.*  Was changed to:  *Meta-analyses were performed for minutes of activity spent as SB, LPA, MPA, MVPA, VPA and daily steps in free-living conditions. A random-effects model was used given the heterogeneous population included. Results were presented in forest plots with overall estimates of the PA level including subgroup-analyses of bouted and unbouted PA. Subgroup-analyses were performed in order to investigate the possible differences in PA arising from analyzing data as bouted versus unbouted, which have been suggested in earlier studies [29–31]. (lines 214-219).*  The following text was added to section 2.6;  *In studies reporting two measures of PA (e.g. bouted and unbouted PA), the number of participants were split in two groups of smaller sample sizes, and results reported as two separate estimates as recommended by Cochrane [23]*. *(lines 172-174)* |
| Results/Discussion: The results found are not properly justified and analyzed in the discussion. There is only a comparison with the existing bibliography. | We thank the reviewer for this comment, which we find very important since a clear overall interpretation and discussion of the results are imperative to us as well as potential future readers of the study. We did however find it challenging to interpret the comment since the reviewer did not refer do any specific parts of the results discussion.  When writing up the manuscript we strived to achieve a balanced but thorough discussion of all results. We have discussed the conflicting results found in other literature *(Hains-Moinfette et al.)* in regards to levels of SB, LPA and MVPA. We also discussed the level of steps by comparing our result to two large and frequently cited studies *(Basset et al. 2010, Althoff et. al 2017)*. We furthermore discussed the conflict between our results, showing a relatively high level of MVPA, compared to results based on large-scale data from the UK biobank *(Chudasma et al. 2019)* that have previously documented that a high proportion of people with multimorbidity are insufficiently physically activity. In these discussions we argued that direct comparisons between our results and other studies of physical activity in people with multimorbidity are very challenging, since different definitions of multimorbidity have been used, and factors such as severity of the conditions are either absent or inconsistently reported. We do acknowledge that certain aspects could be elaborated. We have therefore implemented the reviewers comment by changing the wording and adding text and a reference *(Steeves et al. 2019)* to the results discussion, further highlighting the possible impact of selection bias on the results in the present review.  Last, we wish to highlight that we did discuss results against a range of studies, which were not introduced until the discussion section *(Tudor-Locke et al. 2008, Hains-Monfette et al. 2019, Gimeno-Santos et al. 2014, Basset et al. 2010, Althoff et. al 2017, Vancampfort et al 2017, Chudasma et al. 2019)*. However, should the reviewer still find it necessary for us to elaborate parts of the discussion, we invite the reviewer to provide specific suggestions on which results to discuss further. | The text now reads;  *This appears conflicting to the relatively high daily level of MVPA found in the meta-analysis. Wide variation in PA levels have however also been demonstrated in previous studies of self-reported PA in people with multimorbidity [61] and in the general healthy population [63]. Possible explanations could be the use of different measurement methods (self-reported vs. objectively measured PA) or that selection bias was introduced in the present study and could have affected the estimates. This is likely since the included populations displayed differences in characteristics such as age or specific chronic conditions. Last, functional status of the different populations were unknown since this data was not available or inconsistently reported. If the some of the populations included in the MVPA meta-analyses had higher functional status level, this could have resulted in higher MVPA estimates as supported by previous research [64]. (lines 362-371)* |
| **Reviewer #3 (Dr. Liliana Ricardo Ramos)** |  |  |
| - Change the Cochrane Handbook reference to just Cochrane (page 5, line 83 and others...); |  | Edited according to reviewers suggestion |
| - In the sample collection process, it is indicated that a comparative pilot study was carried out between reviewers. They must indicate the results of this pilot study; | The authors thank the reviewer for the comment. In the present study, no adjustments or changes to coding schemes or eligibility criteria was performed and therefore left out the description since it was considered redundant. The Cochrane Handbook states that it is not recommend using kappa statistics since it is unlikely to convey the real impact of any disagreements in a review (*Cochrane Handbook 7.2.6, Introductory text*). Instead, it is recommended that the reasons for any disagreement be explored since they may reveal the need to revisit eligibility criteria or coding schemes for data collection. In this case, resulting changes should be reported. We however acknowledge that the text can seem ambiguous and have implemented the comment by adding to the text clarifying that no adjustments were made. | The text now reads;  *The extraction procedure was pilot tested on three studies and data compared among the reviewers in order to implement any adjustments to the data extraction form. No adjustments were made. (lines 140-142)* |
| - In table 2, column 8, sometimes they put a control group/intervention group and other times only control/intervention: they must be standardized; | We thank the reviewer for the comment and demonstrating a sharp eye - standardization in tables and text are important. We have changed the wording and added text to the tables in order to clarify which data are related to which groups. | For all studies having more than one group, the word ‘group’ was added in the column ‘Daily physical activity’ in Table 2. We furthermore added group names to describe which data belonged to which groups in table 1.  The abbreviations; EX: Exercise, EUC: Enhanced Usual Care from notes in table 2 since they were not used in this table. They were however added to notes in Table 1 since abbreviations were used here. |
| - The authors begin the discussion of results stating that this is the first systematic review about the level of physical activity in this population - they should not make this statement as they cannot guarantee its veracity...; | We thank the reviewer for this comment and have changed the wording. | The section now read;  *Results from this systematic review and meta-analysis showed that the daily level of MVPA exceeded what is recommended by the WHO for people with chronic conditions, and that the few studies specifically reporting proportions that adhere to PA guidelines found wide variation in adherence (7,4-43%). Results also showed that participants spent on average more than eight hours per day in SB and had a number of daily steps equivalent to a low active lifestyle among adults [13,53].(lines 323-329)* |
| - Authors carry out several expressions like "We did", "we carried out", "our review". These terms must be changed throughout the text to remove orality marks. |  | Personal pronouns have been edited throughout the manuscript according to the reviewer’s suggestion.  Furthermore corrected misspellings throughout the manuscript such as changing behavior (American English) to behavior (British English). |
